# Supplementary figures and images for: Environmental flows or economic woes—Hydropower under global energy market changes
Source: PLoS One. 2020 Aug 5;15(8):e0236730. doi: 10.1371/journal.pone.0236730 (PMC7406062; doi:10.1371/journal.pone.0236730)

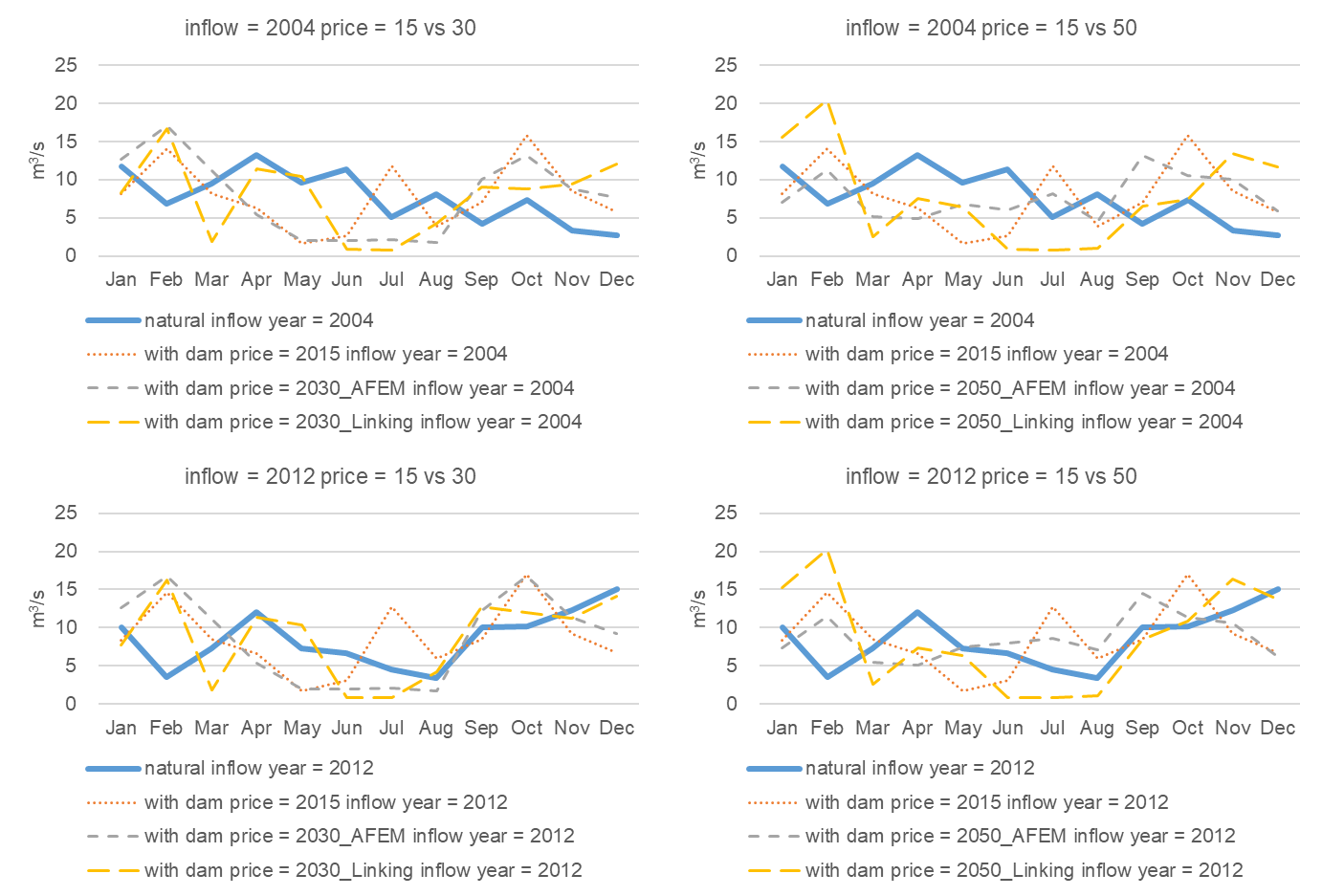

Supplement: S1 Fig — Runoff values are based on the years 2004 and 2012; prices are based on the years 2015, 2030 and 2050. For the prices in 2030 and 2050, two future simulations (AFEM [49] and Link-ing [50] are considered. (TIF) [file pone.0236730.s001.tif]
