# Supplementary material for: Environmental flows or economic woes—Hydropower under global energy market changes
Source: PLoS One. 2020 Aug 5;15(8):e0236730. doi: 10.1371/journal.pone.0236730 (PMC7406062; doi:10.1371/journal.pone.0236730)
Supplement: S1 Table — (DOCX) [file pone.0236730.s002.docx]

|  | **Sense** | **Spöl** |
| --- | --- | --- |
| Catchment area (km^2^) | 352 | 434 |
| Mean runoff (m^3^/s) | 9 | 11 |
| Length (km) | 36 | 42 |
| Elevation distance (m) | 1100 | 1167 |
| Glaciated area (%) | 0 | 0 |
