# Supplementary material for: Environmental flows or economic woes—Hydropower under global energy market changes
Source: PLoS One. 2020 Aug 5;15(8):e0236730. doi: 10.1371/journal.pone.0236730 (PMC7406062; doi:10.1371/journal.pone.0236730)
Supplement: Attachment — Submitted filename: PONE-D-20-04709_reviewer_comments.pdf [file pone.0236730.s004.pdf]

PLOS ONE

Environmental flows or economic woes - hydropower under global energy market changes

--Manuscript Draft--

**Manuscript Number:**	PONE-D-20-04709
**Article Type:**	Research Article
**Full Title:**	Environmental flows or economic woes - hydropower under global energy market changes
**Short Title:**	Environmental flows or economic woes
**Corresponding Author:**	Philipp Emanuel Hirsch<br>University of Basel<br>Basel, SWITZERLAND
**Keywords:**	hydropower; energy transition; flow dynamics; hydropeaking; energy policy
**Abstract:**	The global energy system changes towards renewables-dominated and liberalized markets. This requires making novel trade-offs between the profitable development of hydropower and its environmental effects on the natural flow regime. Here, we use a pristine river as a model for how these future changes will affect the natural flow regime and identify future changes on previously overlooked levels. We identify that damming and discharging based on market prices leads to first- and second-level deviation from natural flows. Beyond these effects, we identified a third level of distance from natural flow. This third level is created by the transition towards a renewables-dominated energy system. The volatile energy input from renewables incentivizes hydropower plant operators to discharge based on more flexible trading behavior. We conclude that novel economic models be combined with tailored implementations of environmental flows. This will allow to find novel solutions for the trade-off between market liberalization and sustainable hydropower development.
**Order of Authors:**	Moritz Schillinger
	Hannes Weigt
	Philipp Emanuel Hirsch
**Additional Information:**	
**Question**	**Response**
**Financial Disclosure**<br><br>Enter a financial disclosure statement that describes the sources of funding for the work included in this submission. Review the submission guidelines for detailed requirements. View published research articles from *PLOS ONE* for specific examples.<br><br>This statement is required for submission and **will appear in the published article** if the submission is accepted. Please make sure it is accurate.	The author(s) received no specific funding for this work.

Powered by Editorial Manager® and ProduXion Manager® from Aries Systems Corporation

**Unfunded studies**<br>Enter: *The author(s) received no specific funding for this work.*<br><br>**Funded studies**<br>Enter a statement with the following details:<br>• Initials of the authors who received each award<br>• Grant numbers awarded to each author<br>• The full name of each funder<br>• URL of each funder website<br>• Did the sponsors or funders play any role in the study design, data collection and analysis, decision to publish, or preparation of the manuscript?<br>• **NO** - Include this sentence at the end of your statement: *The funders had no role in study design, data collection and analysis, decision to publish, or preparation of the manuscript.*<br>• **YES** - Specify the role(s) played.<br><br>* typeset	
**Competing Interests**<br><br>Use the instructions below to enter a competing interest statement for this submission. On behalf of all authors, disclose any competing interests that could be perceived to bias this work—acknowledging all financial support and any other relevant financial or non-financial competing interests.<br><br>This statement **will appear in the published article** if the submission is accepted. Please make sure it is accurate. View published research articles from *PLOS ONE* for specific examples.	The authors have declared that no competing interests exist.

**NO authors have competing interests**<br><br>Enter: *The authors have declared that no competing interests exist.*<br><br>**Authors with competing interests**<br><br>Enter competing interest details beginning with this statement:<br><br>*I have read the journal's policy and the authors of this manuscript have the following competing interests: [insert competing interests here]*<br><br>* typeset	
**Ethics Statement**<br><br>Enter an ethics statement for this submission. This statement is required if the study involved:<br><br>• Human participants<br>• Human specimens or tissue<br>• Vertebrate animals or cephalopods<br>• Vertebrate embryos or tissues<br>• Field research<br><br>Write "N/A" if the submission does not require an ethics statement.<br><br>General guidance is provided below. Consult the submission guidelines for detailed instructions. **Make sure that all information entered here is included in the Methods section of the manuscript.**	N/A

**Format for specific study types**<br><br>**Human Subject Research (involving human participants and/or tissue)**<br>• Give the name of the institutional review board or ethics committee that approved the study<br>• Include the approval number and/or a statement indicating approval of this research<br>• Indicate the form of consent obtained (written/oral) or the reason that consent was not obtained (e.g. the data were analyzed anonymously)<br><br>**Animal Research (involving vertebrate animals, embryos or tissues)**<br>• Provide the name of the Institutional Animal Care and Use Committee (IACUC) or other relevant ethics board that reviewed the study protocol, and indicate whether they approved this research or granted a formal waiver of ethical approval<br>• Include an approval number if one was obtained<br>• If the study involved *non-human primates*, add *additional details* about animal welfare and steps taken to ameliorate suffering<br>• If anesthesia, euthanasia, or any kind of animal sacrifice is part of the study, include briefly which substances and/or methods were applied<br><br>**Field Research**<br><br>Include the following details if this study involves the collection of plant, animal, or other materials from a natural setting:<br>• Field permit number<br>• Name of the institution or relevant body that granted permission	
**Data Availability**<br><br>Authors are required to make all data underlying the findings described fully available, without restriction, and from the time of publication. PLOS allows rare exceptions to address legal and ethical concerns. See the PLOS Data Policy and FAQ for detailed information.	No - some restrictions will apply

Powered by Editorial Manager® and ProduXion Manager® from Aries Systems Corporation

A Data Availability Statement describing where the data can be found is required at submission. Your answers to this question constitute the Data Availability Statement and **will be published in the article**, if accepted.<br><br>**Important:** Stating 'data available on request from the author' is not sufficient. If your data are only available upon request, select 'No' for the first question and explain your exceptional situation in the text box.<br><br>Do the authors confirm that all data underlying the findings described in their manuscript are fully available without restriction?	
**Describe where the data may be found in full sentences. If you are copying our sample text, replace any instances of XXX with the appropriate details.**<br><br>• If the data are **held or will be held in a public repository**, include URLs, accession numbers or DOIs. If this information will only be available after acceptance, indicate this by ticking the box below. For example: *All XXX files are available from the XXX database (accession number(s) XXX, XXX.).*<br>• If the data are all contained **within the manuscript and/or Supporting Information files**, enter the following: *All relevant data are within the manuscript and its Supporting Information files.*<br>• If neither of these applies but you are able to provide **details of access elsewhere**, with or without limitations, please do so. For example:<br><br>*Data cannot be shared publicly because of [XXX]. Data are available from the XXX Institutional Data Access / Ethics Committee (contact via XXX) for researchers who meet the criteria for access to confidential data.*<br><br>*The data underlying the results presented in the study are available from (include the name of the third party*	All relevant data, with exception of the day-ahead market prices, are within the paper and its Supporting Information files. Day-ahead market prices are from EPEX SPOT (https://www.epexspot.com/en), due to copyright issues they cannot published by the authors. However, prices from 2005 to 2015 are also available from https://transparency.entsoe.eu/.

Powered by Editorial Manager® and ProduXion Manager® from Aries Systems Corporation

and contact information or URL).<br>• This text is appropriate if the data are owned by a third party and authors do not have permission to share the data.<br><br>* typeset	
Additional data availability information:	

Click here to download Manuscript Schillinger et al - Environmental flows or economic woes.pdf

Environmental flows or economic woes – hydropower under global energy market changes

Moritz Schillinger[1], Hannes Weigt[1], Philipp Emanuel Hirsch[2*]

1 Research Centre for Sustainable Energy and Water Supply, University of Basel, Basel, Switzerland
2 Program Man-Society-Environment, Department of Environmental Sciences, University of Basel, Basel, Switzerland

* philipp.hirsch@unibas.ch

Abstract

The global energy system changes towards renewables-dominated and liberalized markets. This requires making novel trade-offs between the profitable development of hydropower and its environmental effects on the natural flow regime. Here, we use a pristine river as a model for how these future changes will affect the natural flow regime and identify future changes on previously overlooked levels. We identify that damming and discharging based on market prices leads to first- and second-level deviation from natural flows. Beyond these effects, we identified a third level of distance from natural flow. This third level is created by the transition towards a renewables-dominated energy system. The volatile energy input from renewables incentivizes hydropower plant operators to discharge based on more flexible trading behavior. We conclude that novel economic models be combined with tailored implementations of environmental flows. This will allow to find novel solutions for the trade-off between market liberalization and sustainable hydropower development.

Introduction

Hydropower and Sustainable Development

As the world accelerates towards a new energy system dominated by carbon free renewable energies, novel challenges emerge. These challenges need to be tackled to ensure a sustainable development of our energy supply. Hydropower plays a special role here, because it is a corner stone of our current global electricity provision and its future development will be essential to meet the Sustainable Development Goal (SDG) 7 ‘Affordable and clean energy’ [1]. Hydropower usage always led to trade-offs between energy supply and environmental protection. The anthropogenic discharge dynamics are a major threat to the functioning of natural rivers and wetlands [2] (Fig 1). This functioning relies on the dynamic nature of a natural flow regime [3, 4]. Functioning rivers and wetlands provide essential ecosystem services and are central in achieving the SDGs [5]. Already today, about half of the global river volume is impacted by fragmentation and flow regulation due to hydropower usage [6]. With the global share of undeveloped hydropower remaining high – especially in Asia, Africa, and South America [7–9] – the installed hydro capacity is expected to significantly increase in the coming decades (Fig 1): The IEA World Energy Outlook [8] assumes a total hydropower investment volume of $1.7 trillion until 2040. This enormous future

development is likely to intensify the conflicts between achieving SDG 7 and the SDGs aiming to ensure ecosystem services and biodiversity (i.e. SDG 6 'Clean Water and Sanitation', 14 'Life Below Water', 15 'Life on Land'). A recent study [9] quantified these trade-offs: Globally an unexploited hydropower potential of 5.67 PWh yr^{-1} was estimated. When imposing ecological flow restrictions to protect the natural flow regime (by diverting 30% of the discharge through regulatory mandated flows), this potential is almost halved. This conflict highlights a second, scientifically under-appreciated dimension of conflict: between markets and sustainable development.

New markets, new challenges

The global energy system change will re-shape the way energy is provided and traded. The two major trends of this re-shaping are market restructuring and renewables generation [10]. In the last two decades many electricity systems around the globe were restructured; formerly regulated and monopolized structures were replaced by market competition (i.e. European and American electricity markets, see Fig 1). New actors entered those systems and put pressure on existing structures. Similarly, renewable generation has experienced a significant increase in the last two decades and solar and wind capacities are expected to dominate the future system (e.g. ref. [11, 12], see also Fig 1 for projections up to 2040). Higher shares of wind and solar generation require more flexible plant operation, because their intermittent nature makes electricity production more volatile. In concert, these two developments highlight the role and need for more flexible market structures and point to a gap in our knowledge on the possible environmental ramifications of these developments. Economic research identified the first reactions to these new markets: the emergence of increased short term trading in recent years (i.e. up to 5 minutes before delivery [13]) and new market products such as capacity mechanisms were implemented to ensure sufficient supply in case of low wind and solar injection [14]. Hydropower will be the key element for adapting to these markets by providing the needed flexibility. Unlike wind and solar plants, the operators of storage hydropower plants are able to hold back water and decide when to discharge, thus providing the crucial ability to balance supply and demand [15, 16]. Ecological research has yet to identify how these developments could affect the flow regime of rivers dammed for hydropower beyond the existing models that informed environmental regulations.

Fig 1. Global impact of hydropower on river systems, electricity market structures, and renewable capacity development. A degree of market liberalization of 0% means full regulation while 100% means full competition. Sources: Global flow regime threat from ref. [6]; electricity market structures based on ref. [17]; renewable capacity from ref. [7, 8].

The three levels of hydropower impacts on natural flows

The conflicts between the SDGs on top of the novel market developments calls for integrated scientific approaches. We will need scenarios and tools for decision making on how to sustainably develop hydropower, whilst consolidating possible conflicts and trade-offs [18]. Against this backdrop, we will provide a first analysis whether and how the electricity market of the future will affect river flows. In detail, we will analyze if this adds additional challenges for environmental flow protection, the achievement of the SGDs, and the development of market structures. We first aim to structure the impact of hydropower on flow regimes and the subsequent environmental conditions and incorporate the above described global system changes into a traceable framework. To

that end, we identify first, second, and third-level deviations of anthropogenic flows from the natural flow regime: The first level reflects the fact that, regardless of the system conditions, any hydropower installation (that is not a purely run-of-river system) will lead to the loss of the natural flow dynamics due to damming and storing water for later usage. Even in a stable electricity system, i.e. the regulated ‘old energy system’, this will lead to challenges for environmental protection. On the second level, hydropower will lead to a new flow dynamic as discharge becomes governed by anthropogenic market drivers. This will be especially relevant in newly developing installations, where elaborate environmental flow controls are not yet implemented. With the emergence of renewable energies these market dynamics will be shaped less by conventional generation and their underlying seasonal and yearly dynamic but more by highly fluctuating supply of intermittent generators. Finally, in the ‘new energy system’ with the dynamics imposed by the ongoing market restructuring and increasing intermittent generation, we anticipate a third level of distance from natural flows. This third level reflects a novel market era. In this era, hydropower operators will react to multiple potential drivers aiming to maximize their revenue going from a simple single-market focus (aiming at energy provision) to a multi-market focus (aiming at providing both energy and flexibility). This market superimposition will create a novel anthropogenic forcing on top of the traditional deviations from natural flows. Whilst both the second and third levels, hinge on anthropogenic drivers, we differentiate here between tradition single-market and future multi-markets.

A Central European case study exemplifying the global change

The complexity and diversity of hydropower installations and electricity systems around the globe is boundless. To conceptually analyze the future developments, we therefore need a well-described energy system and environmental background knowledge, such as high-resolution run-off data describing a natural river flow. We found these conditions in a virtual case-study approach in a real-world energy and environment setting: we use the German electricity markets and a well-researched alpine river. Germany can be seen as a testbed for global future developments: it rapidly expands its share of renewables while at the same time restructuring the market environment [10, 19] (see Fig 2). Consequently, the conditions prevailing in recent years in Germany are a looking glass into the future challenges that global electricity systems will face. We couple this market background with flow data from the alpine river Sense as the representative system for natural river conditions. The Sense is a 36 km long river with 9,81 m^3/s mean run-off and one of the few remaining pristine alpine rivers in Central Europe without any anthropogenic influences [20, 21]. Being very well monitored, it will provide the needed reference data (daily discharge data from ref. [22], specifications in the Methods section) for the flow assessments. In terms of its size, the Sense stands representative for the global surge in the development of small hydropower plants (SHP, mostly less than 10 MW although not strict definition exists). A recent global synthesis found that 11 SHP are currently installed for every one large hydropower plant [23]. The global number of 82,891 SHPs is expected to triple and countries like Brazil, China, and Africa are likely to see a leveling off in large hydropower projects whilst SHP development will surge [23–25]. For our analysis, we apply a model representation of hydropower stations developed in ref. [26] and tailor it to the run-off data for the Sense (see the Methods section for details). The relevant outputs are quantifiable targets for both the natural flow regime and the revenue optimal hydropower operation. Following the three-level structure, we implement a storage hydropower station with a dammed river segment and then let it operate according to different market drivers. First, we replicate average system conditions reflecting a situation dominated by either relatively stable regulated structures or markets dominated by mostly conventional power plants.

This will allow us to identify the basic alteration embedded in damming a river (Level 1). Second, we analyze how the virtual Sense hydro station would have operated in the last decades on the German market. This will allow us to get an indication for how market restructuring efforts and new price dynamics imposed by wind and solar may alter flow conditions (Level 2). Third, we will extend the trading behavior of the hydro station by including system service provision as alternative income beside pure energy sales (Level 3). This multi-market strategy represents a more progressive setting, in which not only the generation but also the flexibility of the hydro station is used as an asset. This will allow us to analyze the impact of increasing volatile market conditions on flow conditions in the 'new energy system'.

Fig 2. Historic development of generation from renewable energies, average market prices and market reforms in Germany. Bio= energy from biomass, PV= photovoltaic energy. Sources: Development of renewable energies from ref. [27]; market prices from ref. [28]; and market reforms based on ref. [29].

Material and methods

Hydropower operation model

The hydropower operation model used in this paper is similar to the model described in ref. [26] or ref. [16]. In the model, the hydropower (HP) plant operator maximizes its total revenue R given by the revenue on the respective market m the plant is active on. When having a look at the Level 1 and 2 deviations from the natural flow, the hydropower plant is only active on the electricity market (day-ahead, DA). However, when taking into account trading imposed variations (Level 3), the hydropower plant provides additional system services (here: balancing).

$$\max R = \sum_m R_m \tag{1}$$

In the DA market, energy for delivery at the next day is traded on hourly basis. The revenue on the DA market is the sum of the hourly DA market prices $p_{t,DA}$ and the generation $G_{t,DA}$ on the DA market over a period of one year.

$$R_{DA} = \sum_t p_{t,DA} G_{t,DA} \tag{2}$$

Due to uncertainties or technical problems, imbalances between demand and supply can occur after electricity (day-ahead and intraday) market clearance. However, since demand and supply have to be balanced at all time the Transmission System Operators (TSOs) tender different balancing products to compensate changes in frequency. In this paper only one of these balancing products, namely secondary reserve (SRL), which is tendered on the SRL market, is considered to take into account potential trading imposed variations in the flow (see e.g. ref. [13] for more details on balancing markets). In the SRL market, the provision of capacity as well as the actual energy delivery is remunerated. The capacity $Cap^{+/-}_{t,SRL}$ is remunerated by the capacity price $p^{cap+/-}_{t,SRL}$ and the actual generation $G^{+/-}_{t,SRL}$ is remunerated by the energy price $p^{energy+/-}_{t,SRL}$. In this paper, the German SRL market is taken into account. In the German SRL market weekly asymmetric products are traded. Thus, suppliers can bid separately for the provision of positive and negative balancing capacity on weekly basis (see ref. [30] for details). If a hydropower plants bids for positive SRL, it needs to increase its generation

in times additional energy is required to balance the electricity system $G^{+}_{t,SRL}$. If the plant is active on the negative SRL market, it needs to decrease its generation in times the electricity system is oversupplied $G^{-}_{t,SRL}$.

$$R_{SRL} = \sum_{t} p^{cap+/-}_{t,SRL} Cap^{+/-}_{t,SRL} + \sum_{t} p^{energy+/-}_{t,SRL} G^{+/-}_{t,SRL} \tag{3}$$

The total generation or capacity is constrained by the hydropower plant capacity cap^{max}. If the plant is participating in the upward (positive) SRL market, $Cap^{+}_{t,SRL}$ has to remain free in order to be able to increase generation by the offered capacity level. To participate in the downward (negative) SRL market the hydropower plant needs to run on the DA market at the minimum capacity level cap^{min} plus the capacity which was bid into the negative SRL market $Cap^{-}_{t,SRL}$ in order to be able to decrease its generation in times the electricity system is oversupplied. In our case the minimum capacity cap^{min} is assumed to be zero.

$$G_{t,DA} + Cap^{+}_{t,SRL} \leq cap^{max} \quad \forall t \tag{4}$$

$$G_{t,DA} \geq cap^{min} + Cap^{-}_{t,SRL} \quad \forall t \tag{5}$$

The capacity bid into the SRL market can be called up completely or partly during the underlying period (here: week) by the TSO. Only what is called up ($call^{+/-}_{t,m}$) by the TSO has to be physically produced or reduced by the hydropower plant in the SRL market.

$$G^{+/-}_{t,m} = call^{+/-}_{t,m} Cap^{+/-}_{t,SRL} \quad \forall t, m = SRL \tag{6}$$

The generation or the reduction in generation is given by the water density ρ, the gravity g, the turbine efficiency η, the head H and the discharge $D^{+/-}_{t,m}$. In our case, the head is assumed to be constant.

$$G^{+/-}_{t,m} = \rho g \eta H D^{+/-}_{t,m} \quad \forall t, m \tag{7}$$

The actual discharge D^{net}_{t} at a specific point in time is the difference between the discharge $D^{+}_{t,m}$ and the reduction in the discharge $D^{-}_{t,m}$ in all markets the plant is active on.

$$D^{net}_{t} = \sum_{m} D^{+}_{t,m} - \sum_{m} D^{-}_{t,m} \quad \forall t \tag{8}$$

The discharge can only be reduced by the amount which is discharged through the turbine at a specific point in time.

$$\sum_{m} D^{-}_{t,m} \leq \sum_{m} D^{+}_{t,m} \quad \forall t \tag{9}$$

In addition, the actual discharge is constrained by the maximum d^{max} and minimum d^{min} discharge capacity. The minimum discharge is assumed to be zero.

$$d^{min} \leq D^{\text{net}}_{t} \leq d^{max} \quad \forall t \tag{10}$$

The storage level S_t is defined by the storage of the previous time period S_{t-1}, the utilizable inflows i_t which in our case have been reduced by the minimum residual flow requirements and the discharge. Water can be discharged either through the turbines

(D_t^{net}) or bypassing the turbines ($Spill_t$). However, spilling water would reduce the profit of a hydropower plant since water is not used to generate electricity. If no flow requirements or regulations are in place, the plant operator will not spill any water in order to maximize profit. However, if flow regulations (e.g., Sustainability Boundaries (SDBs) or environmental flows, see below) are in place, spilling water might be necessary to fulfill the requirements.

$$S_t = S_{t-1} + i_t - D_t^{net} - Spill_t \quad \forall t \tag{11}$$

The storage is constrained by the maximum s^{max} and minimum s^{min} storage levels.

$$s^{min} \leq S_t \leq s^{max} \quad \forall t \tag{12}$$

The model is formulated as a linear program and coded in GAMS 24.7.4. The model is solved over a time period of one year using Cplex 12.6 while the smallest time resolution is one hour. The model described above is subject to simplifications and assumptions that can have an impact on the obtainable quantitative results. One major drawback of the model formulation is its deterministic nature. The impact of uncertainty in the water inflows and the prices is neglected. Consequently, the resulting operational decision will represent the theoretic best benchmark given perfect information. Real operational decisions under uncertainty are likely to lead to less optimal behavior of hydropower plants. In addition to uncertainty, technical characteristics of hydropower, e.g. regarding head or turbine efficiency, are simplified in the model formulation. The consideration of more detailed technical characteristics of hydropower could have an impact on the quantitative results. Another limitation of our analysis is the fact that we take the perspective of a single HP plant and assume a perfect competitive market setting. While this is likely true for the electricity market, it may not necessarily hold for balancing markets [26].

Virtual Case Study

In this paper, we rely on a virtual case-study approach. Therefore, a generic hydropower plant is combined with a case of a pristine river.

Generic hydropower plant

In ref. [26] three generic hydropower plants (large, medium, small) which are representative for Switzerland have been defined using the ratio of inflows to storage size and the ratio of storage capacity to turbine capacity as structural indicators. The structural indicators from ref. [26] of the large plant (ratio of inflows to storage size = 2; ratio of storage capacity to turbine capacity = 1000) have been used to define a generic hydropower plant representative for the case study river of this analysis. The derived generic hydropower plant considered in this paper can be classified as a seasonal storage hydropower plant with a storage capacity of 145 Mio. m^3 and a turbine capacity of 175 MW. It represents a typical alpine hydropower project in a high gradient system. While in this paper a generic hydropower plant is considered, the methodology can be applied to any other hydropower plant for which the required data are available. The application of a generic setup with stylized representations like in this paper can both under- and overestimate the real world counterfactuals since hydro plants usually have a high heterogeneity. Focusing on a generic setup therefore only provides an average benchmark when comparing the results to an individual real world plant. However, the model formulation is kept flexible to allow for the inclusion of more detailed plant specific data if it is available [26].

Case Study River

In this paper, the Swiss river “Sense” is used as an example for a pristine river. The “Sense” is one of the most natural river landscapes in Switzerland characterized by a variety of structures, a high consistency and natural fluvial dynamics. The ”Sense” still has intact floodplains, which are classified in Switzerland as being ‘of national importance’; its biodiversity includes i.e. the brown trout (Salmo trutta), a high species-richness of aquatic insects, as well as several amphibian and reptile species endangered in Switzerland [31]. The Sense emerges from the confluence of the “Warmer Sense” and the “Kalter Sense” in the canton of Fribourg. It forms the border river between the canton of Bern and the canton of Fribourg and enters the “Saane”, “Aare”, Rhine and the North Sea. The “Sense” has a length of 36 km, a difference in altitude of 1100 m and a catchment area of 352 km^2 [20]. Daily run-off data for the “Sense” from 1993 to 2018 are available from the Swiss Federal Office for the Environment [22] while the average runoff is approx. 9 m^3/s. In this paper, the years 1993 to 2015 are taken into account regarding the run-off. In order to take the legal flow requirements in Switzerland into account, the run-off of the ”Sense”, which can be used by the hydropower plant, is adjusted by subtracting the minimum residual flow. This residual flow must not be turbinated as stated in the Swiss Water Protection Act (WPA). For additional details on the “Sense” see ref. [20] and ref. [31].

Market Scenarios

In this paper, the German electricity market is used as a case study. In order to compare the “traditional“ hydropower operation with the operation of hydropower plants which is adopted to changes in the electricity system the following two market scenarios are considered:

- single-market
- multi-market

In the “single-market” scenario, the hydropower plant is operated on the electricity (day-ahead, DA) market only. While in the “old” world (here: 2001 to 2003 for Level 1 impacts) electricity prices and dynamics were defined mostly by conventional technologies and stable market structures, the share of renewable energies significantly increased in the past years in the German market while market structures changed (see e.g., ref. [19]). To consider this dynamics the past 15 years (2001 to 2015) are taken into account regarding the DA market (i.e. Level 2 impacts). In the “multi-market” scenario (i.e. Level 3 impacts) the hydropower plant is active on the electricity (day-ahead, DA) and balancing (secondary reserve, SRL) market. Thus, the multi-market scenario takes into account trading behavior of hydropower by including alternative income beside pure energy sales. While there are multiple sources of alternative income, the SRL market is used as an example. Price data which are used in this paper are collected from two sources: hourly day-ahead market prices for Germany are based on ref. [28], SRL market prices are derived from ref. [32]. Regarding the SRL market, only the years 2012 to 2015 are considered. While the SRL market design in Germany changed in 2018, we have a look at the market design which was in place before 2018 (see ref. [32] and ref. [30] for details). Since the SRL capacity prices are based on a pay-as-bid market system, there is no uniform SRL capacity price. Therefore, the volume-weighted mean values were used as SRL capacity prices. In the German SRL market, the SRL energy price and the energy delivery (call up) are based on a merit-order (see ref. [13] and ref. [30] for details). To simulate the merit-order of the German SRL market, the energy price by supplier, the offered capacity and the

called up of energy from ref. [32] are used. However, since we do not know the position of a plant or technology in the merit order, we assume that the HP plant operator bids such an energy price into the SRL market that he/she is called up only if more than two-thirds of the total SRL capacity are required by the TSO.

Sustainability Boundaries and Environmental Flows

The "Sustainability Boundary Approach" (SDA) described by ref. [33] restricts the hydrologic alterations to a range around the natural flow in order to facilitate a suitable water management. Ref. [34] propose that the range for hydrologic alteration should be 10% for a high level of ecological protection and 20% for a moderate level of protection. Thus, the daily flow should not increase or decrease more than 10 or 20% compared to the natural flow. In the analysis at hand, these proposed standards by ref. [34] are taken into account in order to analyze the trade-off between ecological protection and HP operation. The SDBs are defined on the basis of the daily run-off of the respective year, which is simulated. This would require accurate run-off forecasts to define the SDBs in advance. If such forecasts are not available in reality, the SDBs could also be defined on the basis of historical average run-off data. While boundaries on the daily flow are in practice difficult to define [34], monthly flow boundaries are considered as well. To take into account the daily and monthly sustainability boundaries in the HP operation model, two additional constraints are added. The daily or monthly flow out of the reservoir, given by the actual discharge D_t^{net} and the spill $Spill_t$, has to be within the predefined sustainability boundaries SD^{min} and SD^{max} (± 10 or 20% of the natural flow).

$$D_t^{net} + Spill_t \geq SD^{min} \quad \forall t \tag{13}$$

$$D_t^{net} + Spill_t \leq SD^{max} \quad \forall t \tag{14}$$

In addition, the residual flow is not taken into account when analyzing sustainability boundaries since the two requirements are not necessarily complementary. When analyzing environmental flows (flood pulses), only the minimum constraint from above is added to the model. While SDBs constraint the flows at all days or month of the year, the flood pulses are only considered at specific days of the year. The reference data for the flood pulses are based on the historic flood pulses in the river Spöl between 2012 and 2015. To translate the flood pulses of the Spöl to our case study river Sense, we consider the flooding level in the Spöl relative to the average flow conditions in the Spöl. For the timing of the flood pulses we use the same days for the floods as observed in the past in the Spöl (see ref. [22] for daily flows of the Spöl and Sense rivers).

Results and Discussion

Level 1: Negating natural flows

Damming a river is by definition aimed at altering the natural flow pattern and subduing it to the needs of the electricity system. For our example case this is evident from comparing the natural flow variation over two decades with a virtual flow pattern when a dam is installed and operated according to stable electricity system conditions (Fig 3). Evidently, when a dam is built the idiosyncratic natural flow dynamics are lost. We view this as the basic first-level impact of hydropower occurring in regulated electricity systems or markets mostly dominated by fossil and nuclear power plants that are characterized by rather stable operational conditions.

Fig 3. Natural flow pattern vs. standardized flow pattern. Average monthly flow volume for the period 1993 to 2015. The dam is operated according to market prices based on the average hourly prices of the German spot market from 2001 to 2003.

Level 2: Imposing market driven flow variations

With the restructuring of electricity systems and the increasing share of renewable generation in many countries around the globe the formerly rather stable electricity system conditions are increasingly replaced by more volatile market driven conditions. For an example, see the price variation on the German market (Fig 2). Those market conditions in turn influence the operation of hydro plants and thereby the resulting flow pattern. For our example case this impact is evident from the comparison to the natural flow variation (Fig 4). The market dynamic does impose amplitudes in flow that are comparable in scale to a natural variation but not in their seasonality and rising limbs. The rises are less pronounced because they are governed by the turbine capacity in case of a dammed river. The low levels are on even lower levels than on Level 1 because the dam allows to greatly reduce flow volumes for long periods (to benefit from higher prices in later periods). We view these as the Level 2 impacts of hydropower operations as flow alterations become operation-driven. The exact pattern of deviation from Level 1 here is of course highly liable to different market drivers, whose development is uncertain in the future. The development of the 'new energy system' will depend on global fuel prices and carbon policies, the power plant mix of the country in question, renewable and further energy market policies, and electricity demand dynamics. To account for this dependency, we conducted several model runs with different future price scenarios, but there were no fundamental changes in the patterns of change (see Supporting information S1 Fig).

Fig 4. Natural flow variation vs. market driven variation. Based on average weekly flow volumes. The variation of the natural system represents the period 1993 to 2015. The variation of the market driven system is derived by combining the natural inflows from the period 1993 to 2015 with the market price variation in the period 2001 to 2015 (i.e. a matrix of 345 flow/price-year combinations).

Level 3: Adding trading-imposed variations

Finally, as hydro operators adjust to the new markets and optimize their trading structures to benefit from the high flexibility of hydropower, a new dimension of alterations on the river flow patterns is added. We view this as the Level 3 impact of hydropower introducing a novel level of anthropogenic alterations of flows. Fig 5 again shows this for our example case and the difference between a pure energy market oriented trading strategy (i.e. a single market) and an optimized trading behavior aiming at energy markets and system service provision (i.e. multi market in the 'new energy system'). The differences are pronounced with larger variations between peaking and low flow patterns in case of multi market activity. Importantly, the differences here apply in comparison to the level 2 changes in flow regime and not only in comparison to the level 1 or even the natural flow regime. This epitomizes the novelty of this level of change.

Fig 5. Impact of trading activity on flow patterns. Based on average bi-weekly flow volumes for the period 2012 to 2015. The single-market results represent a trading strategy focused on the hourly energy market, the multi-market results represent a strategy allowing trading on energy and system service markets. The shaded area represents the range of the bi-weekly flow volumes.

Trade-offs between following markets and protecting the natural flow regime

Given the desired increase in renewable generation and the respective need to finance new investments and maintain existing plants, we can expect that the Level 3 flow alterations will increasingly become reality for river systems around the globe. Albeit the exact flow pattern will be highly river- and plant-specific the three leveled structure should prevail in general. As the resulting flow pattern tend to lose their natural character the question becomes whether flow regulations could revert this anthropogenic alteration and at the same time maintain sufficient income streams to plant operators to finance their assets. To satisfy the need for quantifiable targets in the development towards such a trade-off, ecologists developed so-called sustainability boundaries (SDB) and the concept of environmental flows [33, 35]. SDBs serve as a framework within which flow alterations are acceptable with the premise of securing the river's ecological integrity as much as possible. SDBs are allowable percentages of deviation from the natural magnitude of flow [33]. To facilitate the cooperation with water managers SDBs were conceived as a hydrologically oversimplified but tangible parameter that can be specifically adapted to any given river [33]. The environmental flows require even more specific tailoring of discharge levels to any given river [35]. Based on ecological research a proxy for a specific ecosystem function of the river is identified and flows are implemented that secure this function [36]. For our analysis, we applied both generic SDBs and transferred a tailored environmental flow regime which had been implemented in a river comparable to the Sense. The generic SBD was a standard set of flow deductions as recommended by ref. [34]. The transferred environmental flows were artificial flood pulses of high un-turbinated discharge. The flood pulses were designed for the river Spöl. The river Spöl is an alpine river flowing in approximately 200 km distance from the Sense. The Spöl was dammed for hydropower. It has a very similar latitude, length, and catchment area than the Sense (see Supporting information S1 Table for a detailed comparison of the rivers). For the sake of our comparison, we assume the restrictions to be implemented at a river stretch that has a comparable mean run-off as the Sense. The flood pulses were designed as environmental flows following a multi-year ecological study. The environmental flow design followed the concept of peak flows flushing fine sediments and benthic algae away to leave clean gravel for gravel-spawning salmonid fish, the brown trout (Salmo trutta), which are socio-ecologically important in many temperate rivers. Research showed that the implementation of the flows did indeed secure the spawning grounds and improve the salmonid's recruitment [37]. To address the trade-offs between securing ecologically relevant flows and economically profitable operation, we statistically tested how the generic SDB would affect the revenue under different scenarios, we used an ANCOVA model with revenue (€-1 MWh-1 week-1) as response variable, time (week) as co-variate, years as random factor, and two categorical predictors: on the one hand the level 3 market scenarios (single and multi) and on the other hand no SDB vs. SDB. As targets for the SDBs we use a 10% and 20% allowed deviation on daily and monthly flows [34]. This is a conservative estimate compared to the 30% which is commonly assumed as an ecological restriction [9]. The daily and monthly differentiation is meant to capture two diverse levels of restrictiveness on operational decisions. The daily

restrictions represent a future in which environmental concerns have priority over economic considerations, whereas the monthly restrictions will allow more flexibility for the operator with arguably more profound effects on the river ecosystem. Overall, the daily SDBs reduce revenues by up to 24% and by up to 7% with the more flexible monthly SDBs (ANCOVA Daily flow deductions: effect of SDB, F=140.89, MS=1.1811, df=1, p=0.0013; ANCOVA Monthly flow deductions: effect of SDB, F=11.04, MS=2.389, df=1, p=0.044, Fig 6). The importance of the market behavior is furthermore evidenced by the significant influence of the scenario (single vs. multi) on the revenue (ANCOVA Daily flow deductions: effect of 'scenario', F=144.36, MS=6.0611, df=1, p=0.0012; ANCOVA Monthly flow deductions, F=55.74, MS=2.1812, df=1, p=0.004). Naturally, with the more restrictive daily SDBs, the flexibility to discharge is so limited for the operator, that the actual market behavior does not make much of a difference in terms of flow patterns. Under the less stringent monthly SDBs, we observe more pronounced difference of the flow patterns between the different market scenarios. To test how specific environmental flow restrictions would impact the revenue, we compared the years 2012 to 2015 with and without artificial flood pulses (the pulses are based on the level and timing of the flood pulses in the river Spöl, see details in the Methods section). Overall the artificial flood pulses affected the revenues to a lesser degree than the generic water deductions (Fig 6). The loss in revenue was largely independent of the actual timing of the artificial flood pulses, indicating some flexibility for the operator to further minimize the water that is lost for turbination. This can be achieved by choosing flood times that serve their ecological function but at the same time occur at times during which the operator is able to spend some water. These exemplary numbers have two major implications: Firstly, they highlight the challenge embedded in environmental flow protection in the increasingly volatile electricity market environment. The restrictions reduce the operator's flexibility to alter the natural flows. As flexibility will have an increasing value in future electricity systems dominated by intermittent renewables, these restrictions tend to increase the resulting economic impact compared to the 'old energy world' with relatively stable electricity system conditions. Secondly, they highlight that an intelligent design of environmental flows can minimize the loss in revenue, whilst potentially safeguarding an important ecological function of the river. On a global scale an unspecific SDB of 30% ecological flow restriction has been estimated to cut the hydropower generation by almost half [9]. Based on such estimates and our analyses it becomes clear that research towards tailored environmental flows is needed to optimize the trade-off between hydropower development and securing the natural river flow in the coming new market future. It should be noted here, that each environmental flow design is specific to a given river. A research program would have to be instigated to specifically design environmental flows for protecting brown trout from adverse hydropower effects in the Sense. Stakeholders, researchers, and political decision makers must work together to identify an ecosystem service, establish a research program and prioritize the installation of environmental flows based on the research. For the river Spöl this is described in detail by ref. [38] and ref. [39] provide a conceptual treatise of such a transdisciplinary approach. Here, the novel implications are that economic modelling of future energy market developments should be an integral part of such research.

Fig 6. Change in average yearly revenue by daily and monthly SDBs and environmental flows (flooding). Based on yearly revenues for the period 2012 to 2015. SDBs represent 10% and 20% allowable deviations from the natural flow on daily or monthly basis. The environmental flows (flooding) are based on the magnitude, frequency, and timing of the environmental flows in the comparable river Spöl.

Conclusion

The challenge for a sustainable future global hydropower development

Our example is a shape of things to come on the global scale. The hydropower of tomorrow will lead to natural flows dynamics that are yet another level distant from natural flows. Although our testbed was located in Europe the processes are relevant to the global hydropower development: with major dam projects, such as the Grand Ethiopian Reservoir Dam, receiving increasing backlash [40], there is a growing trend towards developing small decentralized, carbon-free energy sources [23, 25]. Hydropower in rivers the size of our case-study will be prime targets for such future hydropower development. We acknowledge of course that existing hydropower licenses already frequently have flow requirements that limit operational flexibility of hydropower plants to respond to the grid. These flow requirements may limit ramp rates and discharges and thus the operator's flexibility to respond to the market. Our results here should therefore be put in the context of how such existing regulations will have to be or could be adapted in the novel energy systems of the future. To achieve both the desired increase in carbon free renewable generation (SDG 7) while maintaining the complex ecosystem services that rivers provide9 , environmental flow assessments will need to include more detailed electricity market representations to capture the resulting flow dynamics of hydropower operation. It is insufficient to use past market dynamics to predict future developments. Given the increasing dynamic of the energy market a sustainable development of hydropower must consider the feedbacks between market decisions and environmental impacts. At the same time the sustainable hydropower development must account for both the revenue prospects of hydro operators and the broader economic impacts of hydropower. Our analysis further demonstrates how a research-based approach of environmental flows might be a promising avenue for finding optimal solutions that safeguard a river's ecological integrity and minimize the losses to the hydropower operator's revenue. This highlights the need to find novel combined research approaches that enable an integrated economic and ecosystem development processes. These approaches must equip hydropower operators, environmental managers, and policy makers with river-specific forecasts of how the ecological integrity of a river can be protected by environmental flows that minimize the loss of revenue. Eventually, this will allow for more informed decisions on the optimal trade-off between market development and sustainable development.

Supporting information

S1 Fig. Monthly average flow without and with hydropower dam for different price scenarios. Runoff values are based on the years 2004 and 2012; prices are based on the years 2015, 2030 and 2050. For the prices in 2030 and 2050, two future simulations (AFEM [41] and Linking [42] are considered.

S1 Table. Comparison of basic characteristics of the rivers Sense and Spöl [20, 22, 43].

S1 File. Data. Data on hydropower plant, flows and balancing market.

Acknowledgments

This research is part of the activities of SCCER CREST (Swiss Competence Center for Energy Research), which is financially supported by the Swiss Commission for Technology and Innovation (CTI) under Grant No. KTI. 1155000154. We thank Dr. Rebecca Lordan-Perret, Joschka Wiegleb and Prof. Dr. Patricia Burkhardt-Holm for their valuable inputs and support.

Author Contributions

P.E.H. and H.W. developed the idea and wrote the paper with inputs from M.S.. M.S. and P.E.H. analyzed the data with inputs from H.W.. M.S. performed the modelling with assistance from H.W..

References

1. United Nations. Transforming our world: the 2030 agenda for sustainable development - Outcome document of Summit for adoption of the post-2015 development agenda; 2015. Available from: http://www.un.org/pga/wp-content/uploads/sites/3/2015/08/120815_outcome-document-of-Summit-for-adoption-of-the-post-2015-development-agenda.pdf.

2. Poff NL, Matthews JH. Environmental flows in the Anthropocence: past progress and future prospects. Current Opinion in Environmental Sustainability. 2013;5(6):667–675. doi:10.1016/j.cosust.2013.11.006.

3. Olden JD, Kennard MJ, Leprieur F, Tedesco PA, Winemiller KO, García-Berthou E. Conservation biogeography of freshwater fishes: recent progress and future challenges. Diversity and Distributions. 2010;16(3):496–513. doi:10.1111/j.1472-4642.2010.00655.x.

4. Strayer DL, Dudgeon D. Freshwater biodiversity conservation: recent progress and future challenges. Journal of the North American Benthological Society. 2010;29(1):344–358. doi:10.1899/08-171.1.

5. Ramsar Commission. Scaling up wetland conservation, wise use and restoration to achieve the Sustainable Development Goals; 2018. Available from: https://www.ramsar.org/sites/default/files/documents/library/wetlands_sdgs_e.pdf.

6. Grill G, Lehner B, Lumsdon AE, MacDonald GK, Zarfl C, Reidy Liermann C. An index-based framework for assessing patterns and trends in river fragmentation and flow regulation by global dams at multiple scales. Environmental Research Letters. 2015;10(1):015001. doi:10.1088/1748-9326/10/1/015001.

7. IRENA. Renewable Electricity Capacity and Generation Statistics: June 2018; 2018. Available from: https://www.irena.org/Statistics/View-Data-by-Topic/Capacity-and-Generation/Statistics-Time-Series.

8. IEA. World Energy Outlook 2017. 2017;doi:10.1787/weo-2017-en.

9. Gernaat DEHJ, Bogaart PW, van Vuuren DP, Biemans H, Niessink R. High-resolution assessment of global technical and economic hydropower potential. Nature Energy. 2017;2(10):821–828. doi:10.1038/s41560-017-0006-y.

10. Streimikiene D, Siksnelyte I. Sustainability assessment of electricity market models in selected developed world countries. Renewable and Sustainable Energy Reviews. 2016;57:72–82. doi:10.1016/j.rser.2015.12.113.

11. Mitchell C. Momentum is increasing towards a flexible electricity system based on renewables. Nature Energy. 2016;1(2):6586. doi:10.1038/NENERGY.2015.30.

12. Bogdanov D, Farfan J, Sadovskaia K, Aghahosseini A, Child M, Gulagi A, et al. Radical transformation pathway towards sustainable electricity via evolutionary steps. Nature communications. 2019;10(1):1077. doi:10.1038/s41467-019-08855-1.

13. Ocker F, Braun S, Will C. Design of European balancing power markets. 2016 13th International Conference on the European Energy Market (EEM). 2016; p. 1–6. doi:10.1109/EEM.2016.7521193.

14. Rintamäki T, Siddiqui AS, Salo A. How much is enough? Optimal support payments in a renewable-rich power system. Energy. 2016;117:300–313. doi:10.1016/j.energy.2016.10.058.

15. Barry M, Baur P, Gaudard L, Giuliani G, Hediger W, Romerio F, et al. The Future of Swiss Hydropower: A Review on Drivers and Uncertainties. FoNEW Discussion Paper. 2015;(2015/01).

16. Hirsch P, Schillinger M, Appoloni K, Burkhardt-Holm P, Weigt H. Integrating Economic and Ecological Benchmarking for a Sustainable Development of Hydropower. Sustainability. 2016;8(9):875. doi:10.3390/su8090875.

17. IEA. Re-powering Markets: Market design and regulation during the transition to low-carbon power systems: Electricity Market Series. 2016;.

18. Griggs D, Nilsson M, Stevance A, McCollum D, eds. A guide to SDG interactions: from science to implementation. International Council for Science (ICSU), Paris. 2017;.

19. Markard J. The next phase of the energy transition and its implications for research and policy. Nature Energy. 2018;3(8):628–633. doi:10.1038/s41560-018-0171-7.

20. Hettrich R, Ruff A. Status und Perspektiven nordalpiner Wildflusslandschaften aus naturschutzfachlicher Sicht: FREIHEIT FÜR DAS WILDE WASSER. Die WWF-Alpenflussstudie. WWF Studie. 2011;.

21. Ruffieux I. Europas wertvollster Alpenfluss. Freiburger Nachrichten;2012.

22. FOEN. Hydrological data and forecasts: Stations and data; 2018. Available from: `https://www.hydrodaten.admin.ch/en/stations-and-data.html`.

23. Couto TBA, Olden JD. Global proliferation of small hydropower plants - science and policy. Frontiers in Ecology and the Environment. 2018;16(2):91–100. doi:10.1002/fee.1746.

24. Jonker Klunne W. Current status and future developments of small and micro hydro in Southern Africa. Hydroenergia. 2012;.

25. UNIDO, ICSHP. World Small Hydropower Development Report 2016: United Nations Industrial Development Organization, Vienna, and International Center on Small Hydro Power, Hangzhou;.

26. Schillinger M, Weigt H, Barry M, Schumann R. Hydropower Operation in a Changing Market Environment – A Swiss Case Study. SCCER CREST Working Paper. 2017;WP3 - 2017/07.

27. BMWi. Renewable Energy Sources in Figures: National and International Development, 2017;.

28. EPEX SPOT SE. Market Data: Day-Ahead Auction; 2018. Available from: `https://www.epexspot.com/en/market-data/dayaheadauction`.

29. Kuittinen H, Velte D. Mission-oriented R&I policies: In-depth case studies: Case Study Report: Energiewende;.

30. Consentec GmbH. Beschreibung von Regelleistungskonzepten und Regelleistungsmarkt: Studie im Auftrag der deutschen Übertragungsnetzbetreiber. 2014;.

31. WWF. Factsheet WWF Gewässerperle Sense (BE/FR); 2018. Available from: `https://www.wwf.ch/sites/default/files/doc-2018-03/2018-03-28_Sense_Factsheet.pdf`.

32. regelleistung net. Data Centre: Tender overview; 2018. Available from: `https://www.regelleistung.net/ext/tender/?lang=en`.

33. Richter BD. Re-thinking environmental flows: from allocations and reserves to sustainability boundaries. River Research and Applications. 2010;27:n/a–n/a. doi:10.1002/rra.1320.

34. Richter BD, Davis MM, Apse C, Konrad C. A PRESUMPTIVE STANDARD FOR ENVIRONMENTAL FLOW PROTECTION. River Research and Applications. 2012;28(8):1312–1321. doi:10.1002/rra.1511.

35. Arthington AH, Bunn SE, Poff NL, Naiman RJ. THE CHALLENGE OF PROVIDING ENVIRONMENTAL FLOW RULES TO SUSTAIN RIVER ECOSYSTEMS. Ecological Applications. 2006;16(4):1311–1318. doi:10.1890/1051-0761(2006)016[1311:TCOPEF]2.0.CO;2.

36. Poff NL, Tharme RE, Arthington AH. Evolution of Environmental Flows Assessment Science, Principles, and Methodologies. In: A C Horne, J A Webb, M J Stewardson, B Richter, and M Acreman, editors Water for the Environment. 2017; p. 203–236. doi:10.1016/B978-0-12-803907-6.00011-5.

37. Mannes S, Robinson CT, Uehlinger U, Scheurer T, Ortlepp J, Mürle U, et al. Ecological effects of a long-term flood program in a flow-regulated river. Revue de géographie alpine. 2008;(96-1):125–134. doi:10.4000/rga.450.

38. Scheurer T, Molinari P. Experimental floods in the River Spl, Swiss National Park: Framework, objectives and design. Aquatic Sciences - Research Across Boundaries. 2003;65(3):183–190. doi:10.1007/s00027-003-0667-4.

39. N'Guyen A, Hirsch PE, Adrian-Kalchhauser I, Burkhardt-Holm P. Improving invasive species management by integrating priorities and contributions of scientists and decision makers. Ambio. 2016;45(3):280–289. doi:10.1007/s13280-015-0723-z.

40. Stokstad E. Power play on the Nile. Science (New York, NY). 2016;351(6276):904–907. doi:10.1126/science.351.6276.904.

41. Garrison JB, Demiray T, Abrell J, Savelsberg J, Weigt H, Schaffner C. Combining Investment, Dispatch, and Security Models - An Assessment of Future Electricity Market Options for Switzerland. 2018 15th International Conference on the European Energy Market (EEM). 2018; p. 1–6. doi:10.1109/EEM.2018.8469895.

42. Schlecht I, Weigt H. Linking Europe: The Role of the Swiss Electricity Transmission Grid until 2050. Swiss Journal of Economics and Statistics. 2015;151(2):125–165. doi:10.1007/BF03399415.

43. Wikipedia. Spöl; 2019. Available from: https://de.wikipedia.org/wiki/Sp%C3%B6l.

Click here to download Figure Fig 1.tif

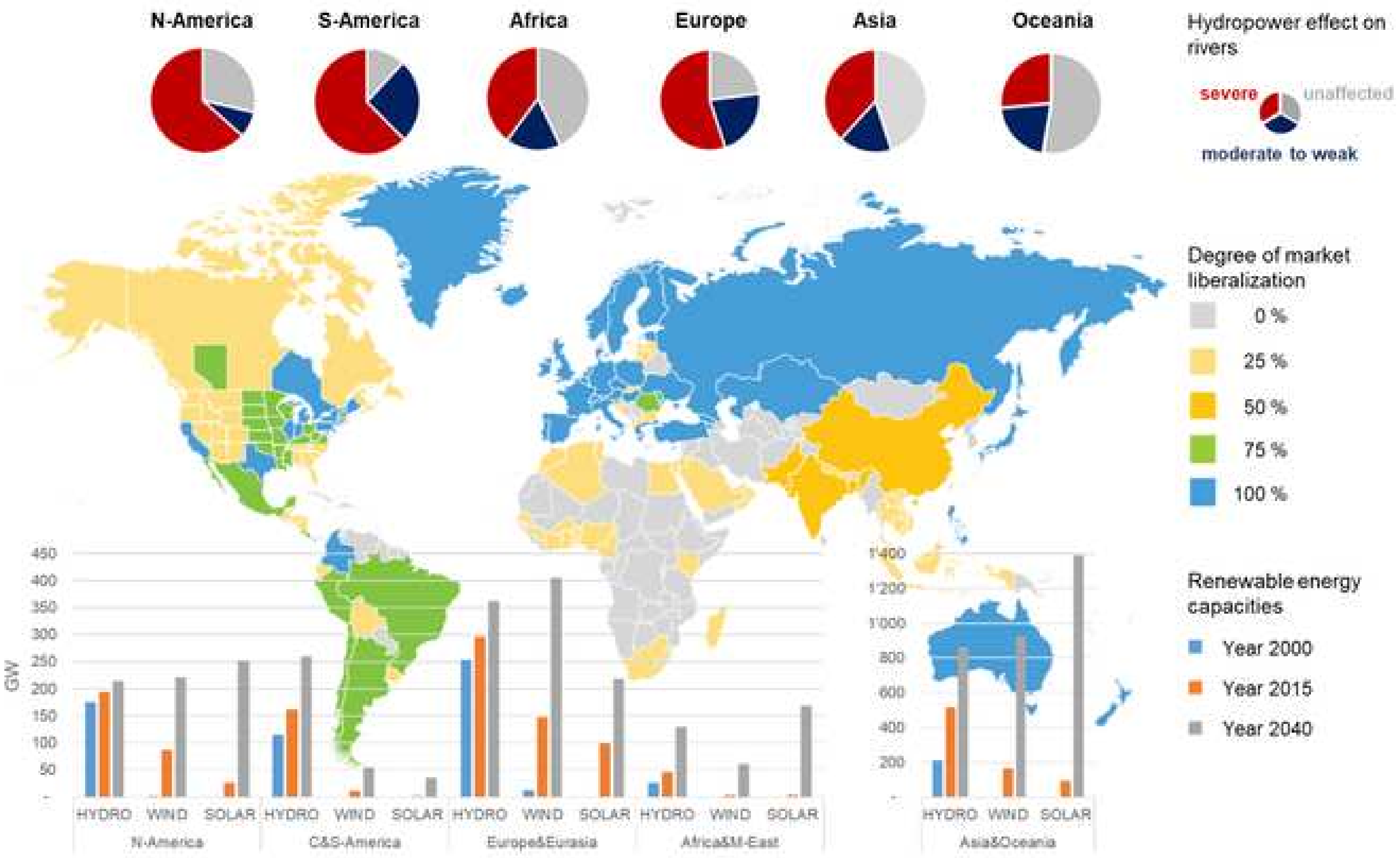

Click here to download Figure Fig 2.tif

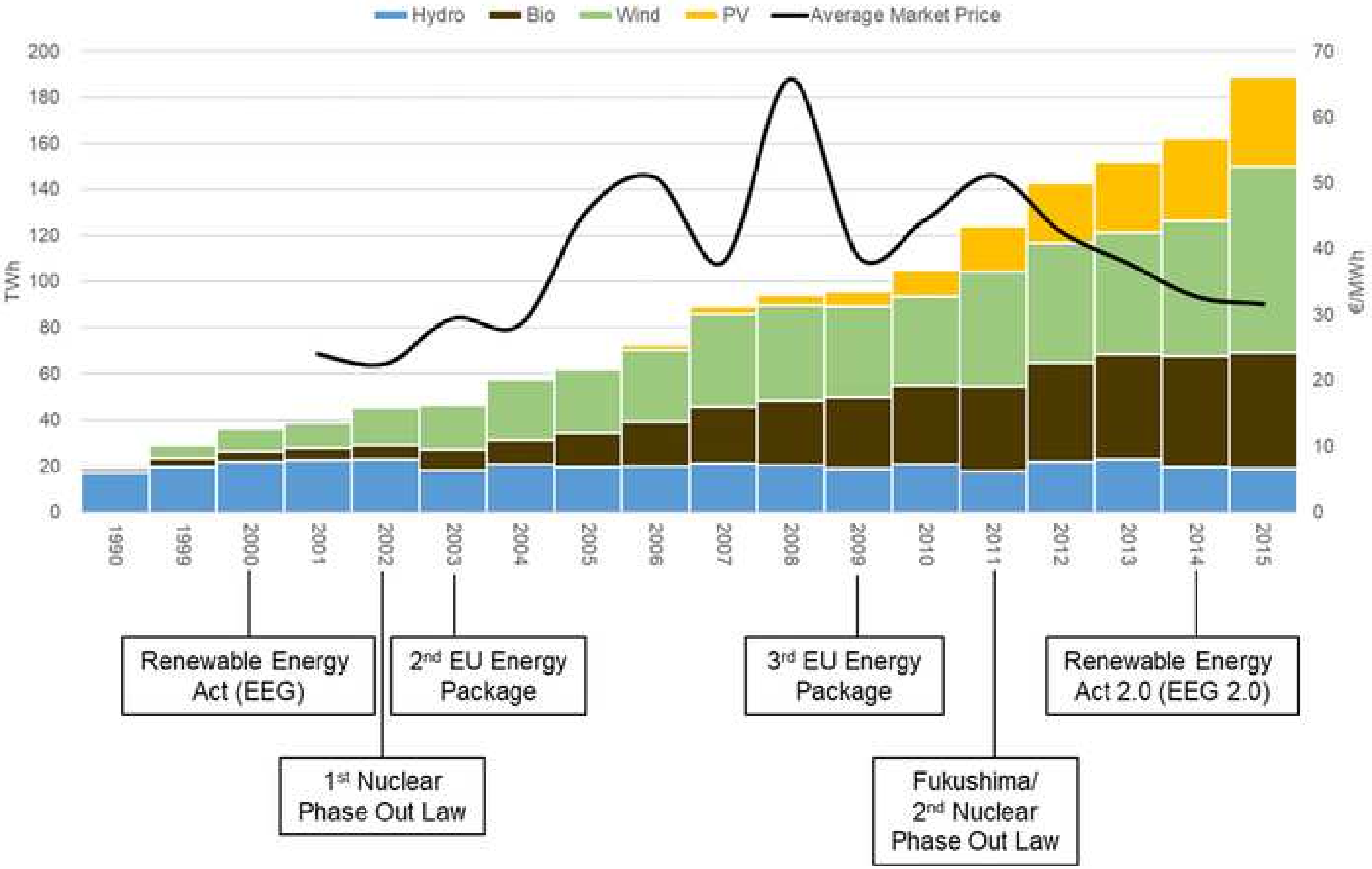

Click here to download Figure Fig 3.tif

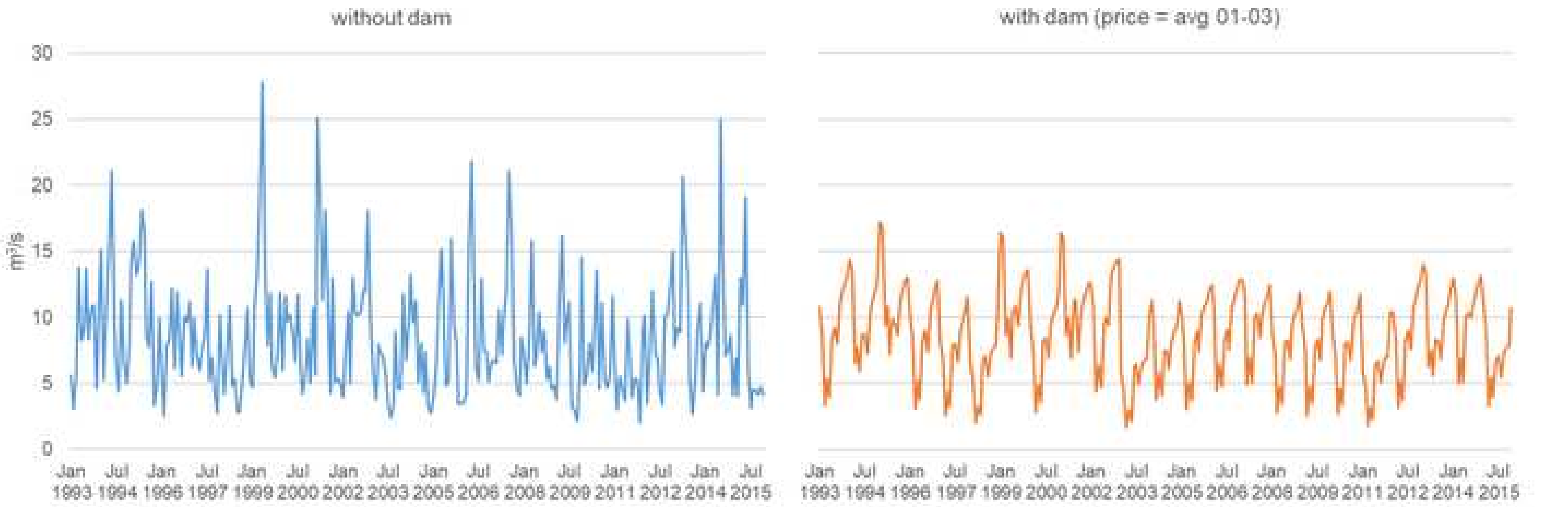

Click here to download Figure Fig 4.tif

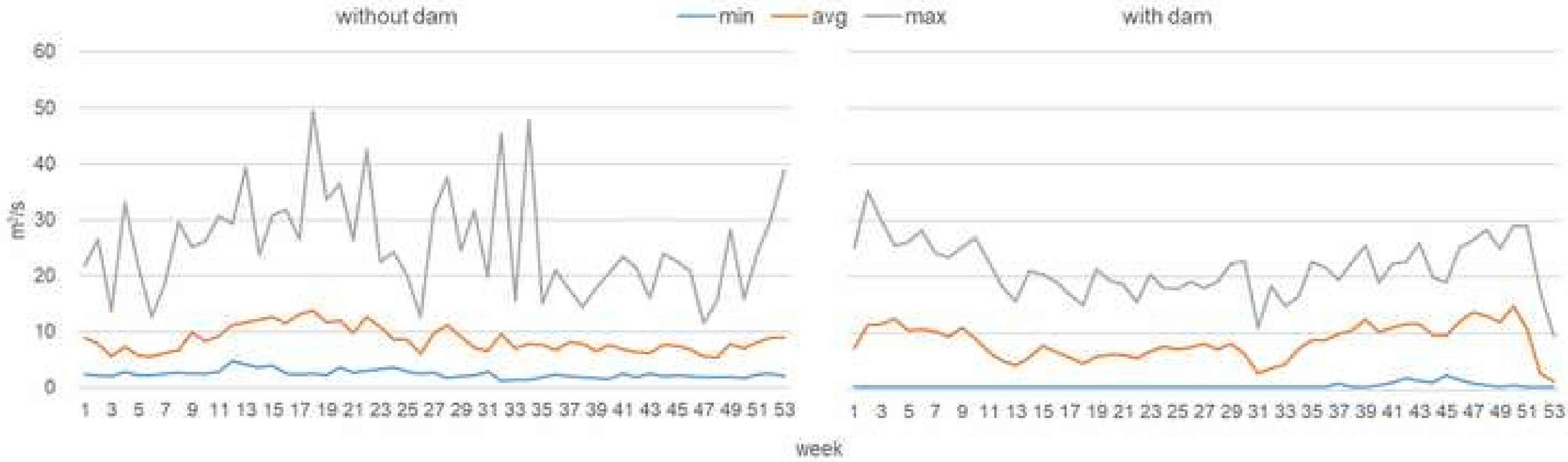

Click here to download Figure Fig 5.tif

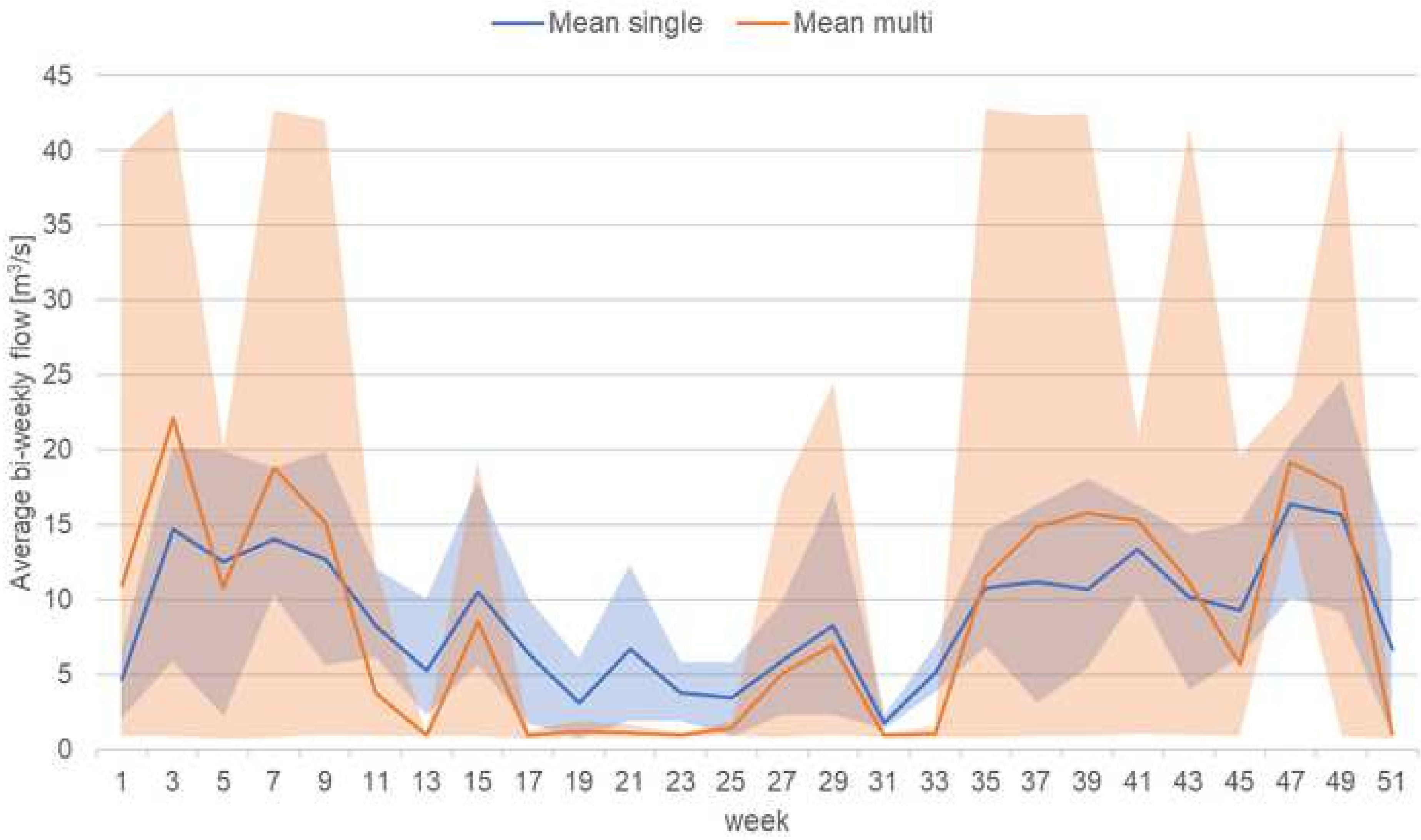

Click here to download Figure Fig 6.tif

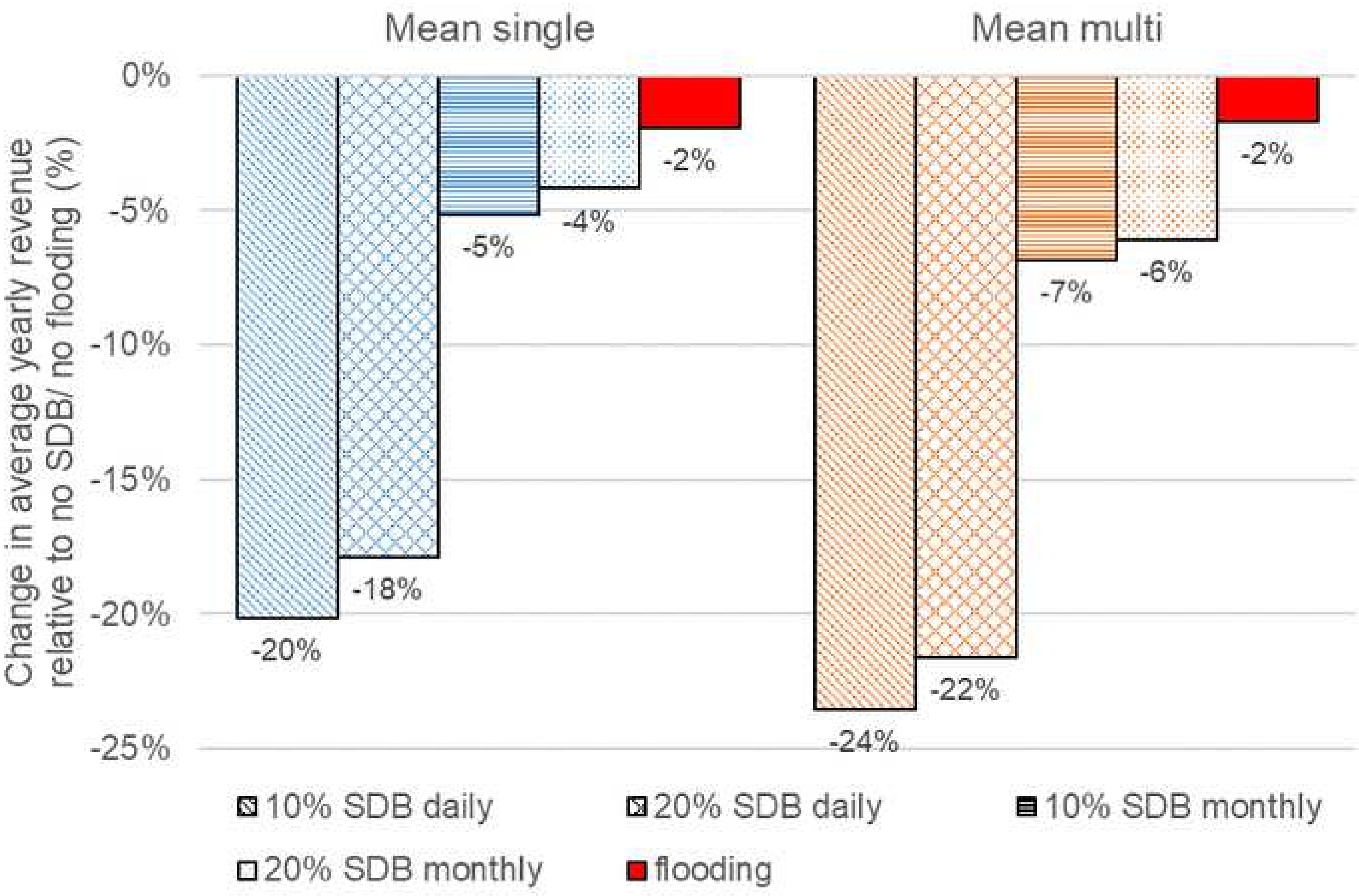

Click here to access/download
Supporting Information
S1 Fig.tif

Click here to access/download
Supporting Information
S1 Table.tif

Click here to access/download
Supporting Information
S1 File.xlsx
