# Supplementary material for: Environmental flows or economic woes—Hydropower under global energy market changes
Source: PLoS One. 2020 Aug 5;15(8):e0236730. doi: 10.1371/journal.pone.0236730 (PMC7406062; doi:10.1371/journal.pone.0236730)
Supplement: Attachment — Submitted filename: Response to Reviewers.docx [file pone.0236730.s005.docx]

| Judi Hewitt  Academic Editor  PLOS ONE |  |
| --- | --- |
|  | |
| Basel, 2020-06-26 | |
| Response to reviewers | |
|  | |

Dear Prof. Hewitt,

Thank you for your patience as we prepared the re-submission of our manuscript entitled “**Environmental flows or economic woes - hydropower under global energy market**

**changes**” (Manuscript Number: PONE-D-20-04709).

Please find below, as indicated in the cover letter our response to reviewers.

We appreciate your attention and we are looking forward to your reply.

Best regards

Philipp E. Hirsch of behalf of all authors

POINT-BY-POINT RESPONSE

**Reviewer #1: General comments**
*The general topic of this manuscript (ms) is of interest. The integration of an economic perspective to research into setting environmental flows would be a valuable and reasonably novel contribution to the literature.*

**Our response:**

We appreciate the referee’s acknowledgement of our work!

**Reviewer #1: General comment:**

*Unfortunately, I have five main areas of concern:*
*A. There are a several methodological clarifications required.*

**Our response:**

We clarified all the aspects brought up by the referee. The more detailed comments (in the pdf, treated below) allowed us to be more specific and more reader friendly in explaining the methodology.

**Reviewer #1: General comment:**

*B. The structure is logical in terms of organisation of sections. However, several important methodological details are placed towards the end of the results and discussion section.*
**Our response:**

We re-wrote and re-arranged the methodological details (also see below in the point-by-point response to the detailed comments in the pdf).

**Reviewer #1: General comment:**

*C. A broader appreciation of the breadth of approaches that have been adopted for environmental flow setting internationally is required through appropriate references to the literature.*

**Our response:**

We substantially broadened our treatise of the concept of environmental flows and included several new references. One of these references, a very recent review published some weeks after our initial submission in February this year, provides a perfect background to the clarification of our contribution. (also see below in the point-by-point response to the detailed comments in the pdf).

**Reviewer #1: General comment:**

*D. The 3-level framework proposed is purely descriptive; and therefore subjective. This framework is devalued by the lack of a quantitative method.*

**Our response:**

The 3-level framework is conceived as a conceptual framework providing important insight for future research. The quantitative method we applied to illustrate our case in point is based on state-of-the-art economic modelling. The third level of novel changes to the flow regime of a river due to novel energy markets is indeed quantified. So we cannot agree that the framework is ‘devalued by a lack of a quantitative mehthod’. If we had presented the 3-level framework without a quantification of the changed hydropower operations, then the referee could have made a point that there is a quantitative method lacking. In the present manuscript however, the presentation of all three level rests on transparent quantifications of river flow.

The quantifications we used for our scenarios of the future of hydropower is an important scientific advancement: existing quantifications of e.g. environmental flows proceed from the assumption that past market dynamics would project into the future and that the hydropower plant operators would discharge and hold back water in the coming decades as they have done in the past. Our message was that it is insufficient to use past market dynamics to predict future developments. We are confident that the combination of conceptual advances and exemplary quantification based on a single case study is a valuable contribution to the state of the art.

**Reviewer #1: General comment:**

*E. Lack of any quantification of differences between downstream flow regimes is an unfortunate omission. There is a vast volume of literature on why particular flow regime metrics would be of interest to flow managers (e.g. minimum flows in specific months for fish spawning or passage).*
**Our response:**

We appreciate the referee pointing in this direction. There are two ways in which we like to respond:

1. We actually provided a visualization of the differences between downstream flow regimes in the manuscript and of several scenarios and additional hydrographs also in the supplementary material (Supporting information S1 Fig). Statistical tests are presented in the manuscript for comparison of effects on the revenue of the operator which is the main focus in our manuscript. Further quantifying differences statistically is of no additional value simply because the hydrographs provided in the main manuscript and in the supplementary material give much more detailed insight than any statistical hypothesis test could give. For example, an omnibus test clearly shows significant differences in downstream flow regimes between the market scenarios (ANCOVA, effect of scenario, F=5.12, MS=9.56, df=4, p=0.012). To make more specific post-hoc hypothesis testing there would be a clear empirical reasoning required what to compare, e.g. minimum flows in a specific season as indicated by the reviewer. Making such specific tests in a absence of empirical reasoning, simply based on assumptions is not the rigorous standard for statistics to which we like to adhere.

2. Indeed, specific flow metrics might be of interest to river management from a conservation perspective. In earlier stages of our project we therefore calculated the indicators of hydrological alteration (IHA) based on Richter et al. 1996 (but see also Gao et al. 2009 for a more recent application). We did actually find some differences depending on whether we assume single or multi-market scenarios in the hydropower plant’s operating- decisions (see preliminary figure and figure caption inserted below). Whilst we found this an interesting preliminary result, we deemed it to indirectly related to our main message (effect of markets on hydropower operation) to be included in the final manuscript. After all, just as outlined under point 1. here: because this is a virtual hydropower plant at a pristine river, we do not have a clear empirical flow target for which a quantification would make sense.

Figure caption: Comparison of indicators of hydrological alterations (to the flow regime of the River Sense IHA following Richter et al. 1996). Each graph shows one group of IHA without dam and with dam for two market model scenarios. A: Magnitude of monthly water conditions (Group 1 of IHA) and timing of annual extreme water events (Group 3) as Julian date. B, D: Magnitude of annual extreme water conditions (Group 2) as mean flow during yearly minima and maxima. C: Frequency of high and low pulses (Group 4) as mean number of all yearly rises and falls. E: Rate and frequency of water condition changes (Group 5) as rise rates: mean of all positive differences between consecutive daily values.

**Reviewer #1: General comment:**

Main issues
*1) Level 1-3 is an interesting concept, but difficult for others to implement due to its narrative nature.*

**Our response:**

See our rebuttal to the same point made in comment ‘D’ above.

**Reviewer #1: General comment:**

*2) The presumptive standard of Richter et al. 2012 is applied. If possible, please give examples where this standard has been applied for a real hydropower operation. Apart from a sentence in the conclusion, there is no mention of legislative requirements/procedures (apart from Swiss minimum flow, and the ms does not explained how that is calculated) that would be applied to implement environmental flows in the real world. It is important to recognize that there has been great debate in the environmental flow setting literature in relation to flow setting methods. Approaches range between the “natural flow paradigm” and the “design-a-flow regime” points of view. See these references for examples discussing various approaches.
a. Acreman, M.C. and Ferguson, A.J.D., 2010. Environmental flows and the European water framework directive. Freshwater Biology, 55(1), pp.32-48.
b. Acreman, M., Aldrick, J., Binnie, C., Black, A., Cowx, I., Dawson, H., Dunbar, M., Extence, C., Hannaford, J., Harby, A. and Holmes, N., 2009, March. Environmental flows from dams: the water framework directive. In Proceedings of the Institution of Civil Engineers-Engineering Sustainability (Vol. 162, No. 1, pp. 13-22). Thomas Telford Ltd.*

**Our response:**

In the revised version we clarified the different approaches to environmental flows and more accurately explain the case study of the environmental flows in the River Spöl (Lines 384-396, line numbers here and henceforth referring to the ‘Clean’ manuscript). We used these existing environmental flows as a testbed for our main question: how would future energy markets affect the establishment of environmental flows and the revenue of hydropower plant operators? Due to some methodological oversights in our original submission (see how we solved this issue in the detailed point-by-point-list below), it was not obvious enough that we never intended to establish and quantify environmental flows for our virtual power plant in reality. The approach to establishing environmental flows rests on appropriate legislative frameworks, environmental impact studies, and flow experimentation (see paper by Vermeulen et al. 2019 and a recent review by Owusu et al. 2020; all newly cited in the revised version, Lines 62-66, 520-540).

**Reviewer #1: General comment:**

*3) Lack of a quantification of differences between downstream flow regimes is an unfortunate omission. There is a vast volume of literature on why particular flow regime metrics would be of interest to flow managers (e.g. minimum flows in specific months for fish spawning or fish passage; higher flows for recreational kayaking at the weekend).*

**Our response:**

This is exactly why we introduced the case study of the River Spöl. Such a case study however, could not just be transferred to the virtual power plant we used for our manuscript. But for our message this was also not necessary, because this is an ecological small scale example and we were interested in demonstrating a novel problem that applies possibly to much more than this one example. Finally, we could not even do this because we would need very elaborate ecological data on the Sense. As can be seen from the many case studies cited by the referee, we would be able to add one more case study but we would not be able to focus on exemplifying an entirely novel and previously overlooked impact that could be relevant to hundreds or even thousands of hydropower projects worldwide (Line 358-372)

**Reviewer #1: General comment:**

*4) Hydro-peaking is given as a key word. The issue of hydro-peaking (rapid rises and falls in river flows that cause fish stranding) can be an important environmental consideration, but this topic is not tackled by the ms. Suggest removal of hydro-peaking from key words.*

**Our response:**

Yes true, we removed these key-words.

**Reviewer #1: General comment:**

*5) This is a multi-disciplinary study. Readers may therefore come from a variety of backgrounds. This situation calls for very careful use of technical terms that may me misinterpreted. Please see comments attached to pdf file for specific examples.*

**Our response:**

We are grateful for this comment and the detailed suggestions in the pdf, which we could use to greatly improve the clarity and readability of our manuscript.

**Reviewer #1: General comment:**

*6) Please see comments attached to pdf file for numerous suggestions, comments and requests for clarification.*

**Our response:**

Thank you for these detailed comments. Below we provide a second list in which we address the comments from the pdf.

**Comments from the pdf:**

**Referee’s comment:**

*Key words: Suggest changes to keywords would be beneficial: flow dynamics usually relates to hydraulics; hydropeaking usually relates to very short term changes in flow (relating in fish stranding). Neither of these are central to the article*.

**Our response:**

Yes true. We changed the keywords to more specifically point to our focus of research. The initially submitted keyword were: hydropower; energy transition; flow dynamics; hydropeaking; energy policy. We replaced the words ‘flow dynamics’ and ‘hydropeaking’. The revised keywords now are: hydropower; energy transition; **market dynamics; sustainable development** boundaries; energy policy.

**Referee’s comment:**

*Abstract: A clearer explanation of “first- and second-level deviation from natural flows” is required to provide meaning to the “third level”*

**Our response:**

Yes thank you, we elaborated on this topic an inserted several clarifications.

**Referee’s comment:**

***Line 8:*** *Trivial English improvement; past tense?*

**Our response:**

Yes, present tense is more appropriate here. We changed the sentence accordingly. Line 8

**Referee’s comment:**

*Line 9: In the hydro-ecological literature "dynamics" is used in relation to hydraulics (depth, velocity, shear stress) whereas "hydrological regime" is used in relation to river discharge (mean flow, seasonality, low flow etc).*

**Our response:**

This is a good point to improve clarification of our manuscript in general. We therefore changed the term dynamics to flow regime. In this specific sentence, we wrote “The anthropogenic changes in the hydrological regime” instead of the “anthropogenic discharge dynamics”. Line 9

**Referee’s comment:**

***Line 48/49****: This statement requires clarification. Why refer to "ecological research" rather than "hydrological research" with respect to flow regimes. Please provide some examples to elucidate your argument. Note there have been many studies on the hydroloigcal impacts of hydropower e.g.: McManamay, R.A., Oigbokie, C.O., Kao, S.C. and Bevelhimer, M.S., 2016. Classification of US hydropower dams by their modes of operation. River Research and Applications, 32(7), pp.1450-1468.*

*and the influence of hydropower on ecological habitat/states e.g.:*

*Null, S.E., Medellín-Azuara, J., Escriva-Bou, A., Lent, M. and Lund, J.R., 2014. Optimizing the dammed: Water supply losses and fish habitat gains from dam removal in California. Journal of environmental management, 136, pp.121-131.*

**Our response:**

We clarified this section by inserting a clearer demarcation between both the hydrological research and the ecological research on hydropower and the novel research that is required (by both disciplines) to account for the novel market developments. We gratefully use the examples provided by the referee to elucidate our argument. The new section now reads: “These development of the energy system have ramifications on hydropower that have yet to be fully acknowledged by researchers. For example, hydrological research has greatly advanced our understanding of how hydropower influences the hydrological regime (e.g. McManamay et al. 2016). Ecological research has greatly advanced our understanding on how the hydrological regime affects the environmental parameters such as fish spawning habitats. However, interdisciplinary research including the economic sciences is an urgently needed further advancement. Such research is needed to identify the impacts that the novel market developments could have on the flow regime of rivers (Null et al. 2014). The results of such research will then allow to account for processes that go beyond the models, that have traditionally informed environmental regulations.“ Lines 48-57.

**Referee’s comment:**

***Line 64****: suggest:* *alteration of the natural flow regime*

**Our response:**

The whole section was re-written to account for the following comment (see below). Lines: 59-71

**Referee’s comment:**

*Line 68: This is not necessarily the case. Depending on location, newly developed installations may have to pass through stringent environmental legislation and planning constraints. They may therefore be subject to considerable environmental flow controls.*

**Our response:**

Yes true. In fact, this comment allowed us to make a crucial point of our manuscript even clearer: that the development of flow controls, even the in the early stages of hydropower planning, needs to acknowledge such existing legislative frameworks, which we elaborate on in the revised version. Lines: 59-71.

**Referee’s comment:**

*Line 81: Whilst this paragraph raises several interesting aspects that would influence future hydropower-induced flow regime alteration, it fails to raise the issue of* *how environmental legislation impacts upon environmental flows dowstream of hydropower dams. For example, the Water Framework Directive in the EU plays a highly influential role in* *governing whether potential hydropower developments proceed and how they may operate. Similar legislation exists elsewhere. This legislation often requires protection of endangered or threatened species, protection of rare habitats, and sometimes consultation with local communities. All of these aspects would relate to environmental flow requirements*.

**Our response:** In the revised version, we elaborate on this issue. We follow the lines of argumentation provided in the referee’s comments and include the legislative aspect. We provide additional info and also include new works (Vermeulen et al. 2019). Collectively, we can now make a clearer case in point for the research gap, that we fill: legislation will also depend on a scientific basis for decision making and therefore research has to provide scenarios for future. Lines: 59-71 and other marked sections.

**Referee’s comment:**

*Line 129: trivial point, but care needed here and elsewhere within the article due to interdisciplinary cross-overs and confusion from phrases with different meanings in different fields. This phrase could easily be confused with "ecosystem services"; a term often used in environmental management.*

*Fisher, B., Turner, R.K. and Morling, P., 2009. Defining and classifying ecosystem services for decision making. Ecological economics, 68(3), pp.643-653.* Also, it is not clear what it meant by "balancing".

**Our response:**

We clarified this point: what was meant with service originally here was the additional advantage of the operator to be able to balance supply and demand by discharging or holding back water based on electricity prices. We re-phrased this point as “provides additional services to the electricity system: the ability to balance supply and”demand by discharging or holding back water (also called “balancing"). By inserting this clarification we now also explain better what is meant by balancing in the revised version. Lines: 150-153

**Referee’s comment:**

***Line 133:*** *English -> on hourly basis*

**Our response:**

This probably referred to the lack of ‘an’ here. We therefore corrected the sentence to ‘on an hourly basis’. Line: 154.

**Referee’s comment:**

***Line 136****: English -> at all time*

**Our response:**

Corrected. Line: 159.

**Referee’s comment:**

***Line 141****: please clarify; capacity with respect to what?*

**Our response:**

We clarified this use of the word capacity. What is meant here is the generation capacity. The sentence then reads as follows: “In order for the TSO to be able to compensate for imbalances in the electricity system at any time, SCR suppliers must reserve their generation capacity for the duration of the contract. This reservation of generation capacity as well as the actual production of electricity are remunerated in the SCR market.” Lines: 165-167.

**Referee’s comment:**

**Line 145: “**weekly” *Important point; m in Equation 1 implies monthly, day-ahead implies daily, weekly is mentioned here, Line 184 says solved over a time period of one year while the smallest time resolution is one hour. Clarification of the time-step on which the calculations are being applied is required. This is important because sub-daily flow fluctuations leading to possible fish stranding is one aspect of environmental flow setting in relation to hydropower, while other aspects might include disruption to the general flow regime (seasonality, low flows, flow variations).*

**Our response:** Thank you for pointing this out. We clarified this in the manuscript.

Here is a short explanation: The model has an hourly time resolution, whereby the model is solved for one year (optimization over all hours of a year). The markets considered in the model differ in their trading time resolution. The day-ahead market implies that electricity is traded the day before actual delivery, with hourly products being traded. In the balancing (SCR) market considered in this paper, weekly contracts are traded. This implies that trading takes place a week in advance and that generation capacity and actual power generation are sold for all hours of a week. The different time resolutions of the markets lead to hourly restrictions, which the power plant has to take into account when it is active on the markets (in case of a weekly market, the restrictions apply to all hours of the corresponding week). The "m" in equation 1 does not mean monthly, but stands for markets (*R_m_* = revenue of each market). Lines 191-201.

**Referee’s comment:**

*Line 158: “assumed to be zero” does this fit with a downstream minimum flow requirement?*

**Our response:** See the responses to the comment on line 172 below.

**Referee’s comment:**

*Line 156: “head is assumed to be constant” but in reality head will vary with water level behind the dam.*

**Our response**: That is true, this is a limitation in our simplified model. In the revised version, we elaborated on this and included more clarifications at the end of the model section.

A brief explanation: In reality, the head varies with the water level and influences the generation efficiency of the hydropower plant. However, the relationship between head (and water level) and efficiency is complex and plant-specific. Since we use a generic hydropower plant in this paper, we lack data on this relationship and we have refrained from generalizing this plant-specific relationship.

Nevertheless, the consideration of a non-constant head may slightly change the discharge of a hydroelectric power plant. The most important criterion for companies to decide when and how much water to discharge, however, remains the market (price) and these are represented in high detail in our model. Since the aim of this manuscript is not to quantify the flow changes exactly, but to show the differences between the three levels, not considering the head effects should only have limited influence. Lines: 233-244.

**Referee’s comment:**

*Line 166: “actual discharge” please clarify this sentence. Which discharge is being referred to? Is this water discharge into the river downstream of the dam? This is confusing given Line 174-175.*

**Our response:** We clarified this in the manuscript by renaming the discharge as turbine discharge, when we actually mean the discharge through the turbines. Turbine discharge does not include spillage or residual flow requirements. The amount of water that enters the river downstream of the dam is the discharge through the turbines, the spillage and the residual flow requirements together. Lines: 108,200, 202

**Referee’s comment:**

*Line 172: “minimal discharge is assumed to be zero” how does this tally with a minimum downstream residual flow requirement?*

**Our response:** Yes, this was indeed not clearly enough phrased. We have adapted the sentence in the manuscript. Here a further explanation: For our case study, we subtract the minimum residual water requirements from the inflows into the dam, which can be used by the hydroelectric power plant to generate electricity. Thus, the hydroelectric power plant cannot use this water. When calculating the flow of the downstream river, the residual water requirements are added up again. Accordingly, the residual water was considered in our approach as if it would bypass the power plant. Because of that, the minimum discharge through the turbine can be assumed to be zero. Lines: 206-207, 211.

**Referee’s comment:**

*Line: 178 “If no flow requirements or regulations are in place, the plant operator will not spill any water in order to maximize profit “**But spill may occur during high inflow periods (floods) when the dam is full since it does not have water storage capacity.*

**Our response:** That's true, we made that clear in the manuscript. In general, in the model, the hydropower plant operator can always choose the option of spilling. Therefore, if the dam is full and additional inflows cannot be stored or discharged through the turbine, the power plant would chose to do so. What we meant was that only if the power plant reaches its limits, spilling would take place. However, under normal conditions, the power plant would not use spill, because it would be a lost profit. Line: 211-214.

**Referee’s comment:**

*Line 179: This is very confusing for the reader since neither of these terms has been defined. Suggest delete*

**Our response:** Yes indeed. We deleted this in the revised version.

**Referee’s comment:**

*Line 183: “The model is	solved over a time period of one year”*

*This is confusing since results show the model has been run for several years.*

**Our response:** We have made this clear in the manuscript. In general, our model has an hourly time resolution and is solved over a time horizon of one year. Thus, each year that we consider in the manuscript is solved individually, inter-annual dependencies are not considered endogenously. Lines: 224-226.

**Referee’s comment:**

*Line 235: “...adjusted by subtracting the minimum residual flow.“. This is very important, but few details are provided. What is this minimum residual flow? How it is calculated? Is it a constant flow? Are temporal variations required to incorporate seasonal variations or flushing requirements or recreational requirements as part of this residual flow?*

**Our response:** We have made this clear in the manuscript. See comment above on how we take into account the minimum residual flow in the model. Since we take a Swiss river as a case study, we consider the minimum residual flow requirement in Switzerland based on the Swiss Water Protection Act. The minimum residual flow is defined as the minimum flow of a river that must remain after one or more water withdrawals or damming. It is a constant flow rate defined on the basis of Q347, i.e. the average flow rate reached or exceeded on 347 days a year. Depending on the flow rate Q347, the Swiss Water Protection Act then defines the minimum flow rates as follows:

While in practice a 10-year average of Q347 is used to define the minimum flow requirements, we define Q347 individually for each year we consider (1993-2015). We do this because we have no data before 1993, so we cannot use the 10-year average for the years before 2002. To be consistent, we have therefore defined Q347 on the basis of the year in question for all the years considered. Using the year-specific Q347 value instead of the 10-year average can lead to an over- or underestimation of the minimum residual water requirements in a given year. Over all years considered, the average Q347 value for our case study River Sense is approximately 2500l/s (max: 3000, min: 1400), resulting in an average minimum residual water requirement of 900l/s (max: 1010, min: 570). Lines: 282-298.

**Referee’s comment:**

*Line 236: English? Please define? "must not pass through dam turbines" or "must not be used to generate power"?*

**Our response:**

The section was re-written in the revised version following the comment above.

**Referee’s comment:**

*Line 250: “2005-2015” So only a 3 year sample for the "old" and a 15 year sample for the "mulit-market". Is the difference is period length important in relation to inter-annual variability in climate. Are there any drought conditions or low snow years that make some years different to others with respect to water availability and electricity generation*.

**Our response:** We clarified this in the manuscript. In terms of prices, the years 2001 to 2003 were considered as "old" world (average from 2001 to 2003). To reflect the changes in market dynamics (level 2), the prices for the years 2001 to 2015 were taken into account. For the multi-market setting (level 3) the years 2012 to 2015 were analyzed (only four years due to data availability and the long calculation time when several markets are considered). For hydrological conditions (i.e. runoff), the years 1993 to 2015 are considered. In the case of Level 1 (old world) a cross combination of one average price year (average from 2001 to 2003) and 23 weather years was simulated. In case of Level 2 a cross combination of 15 price years and 23 weather years was considered (345 cases). For Level 3 (multi-market setting) only the runoff for the years 2012 to 2015 was considered. Changes or extremes in the hydrological conditions should therefore be sufficiently taken into account. Lines: 312-321.

**Referee’s comment:**

*Line 263:* *“volume-weighted mean values were used as SRL capacity prices “. The method is not reproducible from the information provided. Please explain further.*

**Our response:** We clarified this in the manuscript. Here a further explanation: On the German SCR market, all participating suppliers submit a price quantity bid, while all suppliers that are accepted on the market are remunerated with their bid price. There is therefore not a single price on the market, but several. To derive a single price for our simulations, we take the average of all accepted prices, weighting the individual prices with the supplier's quantity. Lines 330-338.

**Referee’s comment:**

*Line 272: It is important to note that these two references suggest a hypothetical environmental flow standard. To the best of this reviewers knowledge this standard has not been applied in reality.*

*It is also important to note that Ref 34 states: Our presumptive standard is intended for application only where detailed scientific assessments of environmental flow needs cannot be undertaken in the near term.*

**Our response:** Yes true, we thank the referee for this specification. We included this in the revised version, thus further clarifying how we apply the “Sustainability Boundary Approach" in our case study. Lines: 343-350, 358-360

**Referee’s comment:**

*Line 308/309 / Figure 3: Please clarify that this was a period* *dominated by fossil and nuclear characterizing stable operational conditions.*

**Our response:** We clarified this in the revised version’s figure caption. Lines 432-433.

**Referee’s comment:** *Line: 313 “see the price variation on the German market”* *This may be true, but average market price data from Figure 2 is inconclusive.*

**Our response:** We have generalized this reference to Figure 2. Figure 2 shows the restructuring and increases in renewable energy described at this point using the example of Germany. The average market prices are, however, only of limited use here to illustrate the more volatile market conditions, as electricity prices are influenced by numerous factors (e.g. electricity demand, generation technologies, fuel prices, CO2 prices). Accordingly, there are other factors besides the share of renewable energies or the structure of the electricity system that influence the price level. Lines: 308-311.

**Referee’s comment:**

*316-323: This section is poorly expressed. Please clarify.*

**Our response:**

The section was re-written and now contributes to a clearer demonstration of the proposed 3-level framework.

**Referee’s comment:**

*Line 353 “ ecologists developed so-called sustainability boundaries (SDB) 353*

*and the concept of environmental flows” Important point: this statement ignores a large body of literature describing various approaches to environmental flow setting. One example of an alternative approach is by adaptive management. See:* *Stoffels, R.J., Bond, N.R., Nicol, S. (2018) Science to support the management of riverine flows. Freshwater biology, 63(8), pp.996-1010.*

**Our response:**

Yes of course, this is an important point. We are absolutely aware that there are various examples of more specific environmental flow setting. This is also what we intended to demonstrate by including the river Spöl in our study, because the environmental flows in the river Spöl are a good example for a more specific calculations of environmental flows. In the revised version we clarify this: we insert more explanations for the generic approach we followed by exemplifying the SDBs and the more specific approach by exemplifying the environmental flows of the river Spöl. We also cited the reference provided by the referee. Finally, it is important to emphasize here, that it was not the intention of our study to calculate a specific environmental flow for a virtual hydropower dam, but rather to use a virtual hydropower dam as a looking glass into the energy system future and how this future should be met with more interdisciplinary research. Lines 384-4032

**Referee’s comment:**

*Line 359: “adopted” adapted?*

**Our response:**

Yes, corrected to adapted. Line: 303.

**Referee’s comment:**

*Line 363: “a tailored environmental flow regime which had been implemented in a river comparable to the Sense”. This statement does not match with my understanding of what was given in the methods section.*

**Our response:**

There must have been a misunderstanding. This was only an example of an already existing tailored environmental flow regime, which we here merely use for demonstration purposes. We demonstrate, using an existing example, how relevant our 3-Level-concept is for future development of hydropower and environmental flows. We clarified this in the revised version and also inserted more clarifications on environmental flows and how relevant our 3-Level-concept based on more references (as also suggested by the referee above). Lines: 384-424.

**Referee’s comment:**

*Line 365-377:* *This is important information needed when interpreting the results. Please move to methods section. Also, patterns in snowfall and ice melt can be highly influential for flow regimes in alpine settings. Are these these catchments similar in terms of snowfall and ice melt. Are they at the same altitude?*

**Our response:**

Thank you for the suggestion, we moved the section into the Materials and Methods section and also included some more clarifications based on the other comments. Lines: 342-384

Concerning the questions about the catchments of the two rivers (Spöl and Sense): yes the catchments are similar in terms of snowfall and ice melt, which becomes evident from the percentage of glaciation of the catchment. This is 0 for both catchments. The information can be taken from Swiss Office for the Environment and we included this info in the revised version in the Supplementary Table S1.

**Referee’s comment:**

*Line 378 ff: Please move to methods section*

**Our response:**

Moved to method section, see our response to above comment.

**Referee’s comment:**

*Line 393: What was the replicate number (n) for daily and monthly?*

**Our response:**

Thank you for noticing this oversight, which we corrected in the revised version: The resulting replicate number n per each of the ANCOVAs was 212. The number 212 consists of 53 weeks per each of the 4 years (2012-2015) for which the comparison of weekly revenues was calculated in both the daily and monthly scenario. Line: 408-410

**Referee’s comment:**

*Line 402: Please move to methods section.*

**Our response:**

Thank you for this suggestion. We chose to keep this sentence in place because it provides important background information that the reader should see directly here in combination with the results. We are confident that the revised methods section provides sufficient clarifications and that this sentence here should enhance readability.

**Referee’s comment:**

*Line 408: “turbination”: generation*

**Our response:**

Corrected. Line: 343.

**Referee’s comment:**

*Line 427: References to a large body of literature are missing here. Please see for examples your ref 36 and other chapters of: A C Horne, J A Webb, M J Stewardson, B Richter, and M Acreman, editors Water for the Environment. 2017; p. 203{236. doi:10.1016/B978-0-12-803907-6.00011-5.*

**Our response:**

Thank you for mentioning this. In the revised version, we elaborate on the process of finding environmental flows also by citing more relevant literature. More specifically, we refer to a review which was just recently published by Owusu et al. 2020 (River Research and Applications). This review lays out the processes behind finding environmental flows and provides a fitting synthesis. This synthesis is a perfect opportunity for us to clarify the research gap, which we have filled and the future avenues for research that our study opens up. We also include in this section two new sentences on the legislative aspect, which the referee emphasized to be important in earlier comments. Lines: 537-543.
